# (Na,□)_5_[MnO_2_]_13_ nanorods: a new tunnel structure for electrode materials determined *ab initio* and refined through a combination of electron and synchrotron diffraction data

**DOI:** 10.1107/S2052520616015651

**Published:** 2016-12-01

**Authors:** Enrico Mugnaioli, Mauro Gemmi, Marco Merlini, Michele Gregorkiewitz

**Affiliations:** a Department of Physical, Earth and Environmental Sciences, University of Siena, via Laterina 8, 53100 Siena, Italy; b Center for Nanotechnology Innovation@NEST, Istituto Italiano di Tecnologia, Piazza San Silvestro 12, 56127 Pisa, Italy; c Department of Earth Sciences, University of Milan, via Botticelli 23, 20133 Milano, Italy; d ESRF, European Synchrotron Radiation Facility, 6 Rue Jules Horowitz, 38000 Grenoble, France

**Keywords:** octahedral molecular sieves, cation intercalation electrode material, electron diffraction tomography, dynamical refinement, Rietveld refinement

## Abstract

(Na_*x*_□_1 − *x*_)_5_[MnO_2_]_13_ has been synthesized with *x* = 0.80 (4), corresponding to Na_0.31_[MnO_2_]. This well known material is usually cited as Na_0.4_[MnO_2_] and is believed to have a romanèchite-like framework. Here, its true structure is determined, *ab initio*, by single-crystal electron diffraction tomography (EDT) and refined both by EDT data applying dynamical scattering theory and by the Rietveld method based on synchrotron powder diffraction data (χ^2^ = 0.690, *R*_wp_ = 0.051, *R*_p_ = 0.037, *R*_*F*2_ = 0.035). The unit cell is monoclinic *C*2/*m*, *a* = 22.5199 (6), *b* = 2.83987 (6), *c* = 14.8815 (4) Å, β = 105.0925 (16)°, *V* = 918.90 (4) Å^3^, *Z* = 2. A hitherto unknown [MnO_2_] framework is found, which is mainly based on edge- and corner-sharing octahedra and comprises three types of tunnels: per unit cell, two are defined by S-shaped 10-rings, four by egg-shaped 8-rings, and two by slightly oval 6-rings of Mn polyhedra. Na occupies all tunnels. The so-determined structure excellently explains previous reports on the electrochemistry of (Na,□)_5_[MnO_2_]_13_. The trivalent Mn^3+^ ions concentrate at two of the seven Mn sites where larger Mn—O distances and Jahn–Teller distortion are observed. One of the Mn^3+^ sites is five-coordinated in a square pyramid which, on oxidation to Mn^4+^, may easily undergo topotactic transformation to an octahedron suggesting a possible pathway for the transition among different tunnel structures.

## Abstract 2

Octahedral molecular sieves (OMS) attract increasing interest in the search for novel electrode materials for energy storage and water desalination. While a nanometric particle size is desirable for such applications, this makes ordinary single-crystal characterization difficult and many OMS structures are still waiting for elucidation. Here we present the long awaited structure of a well known material, (Na,□)_5_[MnO_2_]_13_, resolved by a combination of electron diffraction tomography, dynamical scattering theory and X-ray powder Rietveld refinement. A new type of tunnel structure was found, able to explain previously reported electrochemical properties. This structure also suggests a possible mechanism for topotactic transformations between different manganese oxide OMS frameworks.

## 1. Introduction   

(Na,□)_5_[MnO_2_]_13_ belongs to an emergent group of compounds which is now usually referred to as octahedral molecular sieves (OMS; Suib, 2008 ▸), in allusion to their open framework structures resembling zeolite molecular sieves, the well known tetrahedral counterpart. Zeolites are widely used in chemical processes (ion exchange, shape selective catalysis, semipermeable membranes *etc.*; Breck, 1974 ▸; Gorgojo *et al.*, 2008 ▸) and their unique properties can be explained in terms of crystal structure: there are presently 231 different framework topologies (*cf.* http://www.iza-online.org) and efforts are ongoing to find new frameworks and applications (Cundy & Cox, 2003 ▸; Camblor & Hong, 2010 ▸; Bellussi *et al.*, 2012 ▸).

The most promising features which distinguish OMS frameworks are with regard to their electronic properties. While zeolite frameworks are typically electronic insulators, the octahedrally coordinated elements in OMS structures (mostly transition elements from Ti to Co and their homologs) have easily accessible 3*d* (4*d*, 5*d*) orbitals and many different oxidation states may occur.

The title compound (Na,□)_5_[MnO_2_]_13_ (Tsuda *et al.*, 2003 ▸; Hu & Doeff, 2004 ▸; La Mantia *et al.*, 2011 ▸; Liu *et al.*, 2011 ▸), along with other binary or ternary manganese oxides (Doeff, 1996 ▸; Wei *et al.*, 2011 ▸; Lee *et al.*, 2014 ▸; Yabuuchi & Komaba, 2014 ▸; Wang *et al.*, 2015 ▸; Fang *et al.*, 2016 ▸), have recently attracted much interest for their use as electrodes in batteries or in supercapacitors for energy storage or capacitive water desalination, but its properties were so far little understood. Since its first synthesis by Parant *et al.* (1971 ▸), (Na,□)_5_[MnO_2_]_13_ has been assumed to be based on the romanèchite framework, □_2_[MnO_2_]_5_, which exhibits large rectangular 2 × 3 tunnels confined by walls of double and triple octahedral chains (for an exhaustive presentation of these tunnel structures see Pasero, 2005 ▸). In this structure, there is only one crystallographically distinct site for the channel cations, but electrochemical results (Tsuda *et al.*, 2003 ▸; Hu & Doeff, 2004 ▸; Liu *et al.*, 2011 ▸) clearly show 3–4 peaks and plateaus for cation insertion–desorption during charge–discharge and cyclic voltammetry experiments, difficult to reconcile with the expected behaviour of a romanèchite framework.

Parant *et al.* (1971 ▸) already mentioned that several lines in the diffraction pattern of (Na,□)_5_[MnO_2_]_13_ were incompatible with the side centring of the romanèchite unit cell found by Wadsley (1953 ▸). Later, Hu & Doeff (2004 ▸) mention that they were unable to simulate the observed diffraction pattern using the monoclinic unit-cell parameters of Parant *et al.* (1971 ▸) and the romanèchite atom parameters of Turner & Post (1988 ▸).

We found (Na,□)_5_[MnO_2_]_13_ in a more general study about the formation of Na_*x*_MnO_2_ compounds and, as usual with OMS materials, we invariably obtained fine-grained powders made up of needles of < 4 µm in length and 30–60 nm in thickness. While a high degree of dispersion is desirable for most applications, this precludes ordinary single-crystal work to establish the crystal structure. In addition, impurity phases are generally present and make work with these powders difficult.

In the present study, we could overcome this problem using the recently developed (Kolb *et al.*, 2007 ▸, 2011 ▸) electron diffraction tomography (EDT) technique which allows the collection of quasi-kinematical three-dimensional electron diffraction data sets on crystals of a few hundreds of nanometres or smaller. The technique has been used successfully for solving the structure of a variety of nanocrystalline materials (Mugnaioli & Kolb, 2013 ▸; Mugnaioli, 2015 ▸). Here, EDT data collected on selected single needles allowed us to conduct a single-crystal *ab* *initio* structure determination and, in a second step, to undertake a full parameter refinement based on the dynamical theory of diffraction using the methodology recently established by Palatinus *et al.* (2013 ▸), Palatinus, Corrêa *et al.* (2015 ▸) and Palatinus, Petříček & Corrêa (2015 ▸). The model yielded by EDT was independently refined using the Rietveld method based on synchrotron radiation (SR) data, allowing us to establish the chemical formula (Na_*x*_□_1 − *x*_)_5_[MnO_2_]_13_, *x* = 0.80, along with a refined model about Mn^3+^–Mn^4+^ order and the distribution of Na in the channels.

In the last section of this study, the electrochemical properties of (Na,□)_5_[MnO_2_]_13_ are extensively discussed on the basis of the new structure and compared with other tunnel structures (including romanèchite proper), in the perspective of the development of novel OMS materials.

## 2. Experimental methods   

**Figure 1:**
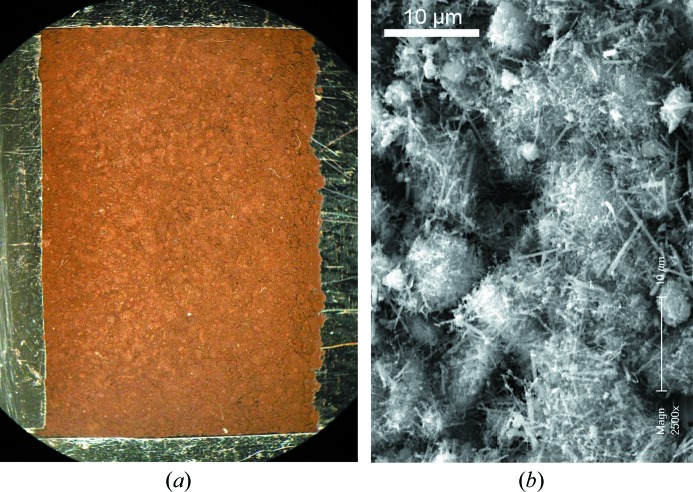
The product obtained at 778 K/24 h. (*a*) Sample as prepared in a silicon holder for Bragg–Brentano X-ray diffraction (11 × 17 mm). (*b*) SEM microphotograph showing needles of (Na,□)_5_[MnO_2_]_13_ and some flakes of Na_2_Mn_3_O_7_/birnessite.

(Na,□)_5_[MnO_2_]_13_ was prepared in a two-step procedure similar to that used by Lan *et al.* (2011 ▸) for the synthesis of manjiroite (Na-hollandite). A solution of 0.8 g NaOH in ∼ 20 ml deionized and freshly boiled water is added slowly, using a magnetic stirrer, to a solution of 1.97 g of MnCl_2_·4H_2_O in ∼ 30 ml of deionized water. The brown precipitate is filtered and washed with deionized water until the effluent reaches pH = 7 and subsequently dried at 363 K for 24 h. For the second step, a small quantity (0.1–0.2 g) of the dry powder is mixed with 4 g NaNO_3_ and heated in a porcelain crucible at 778 K for 24 h. The product of this reaction, mainly (Na,□)_5_[MnO_2_]_13_, is a dark brown powder (Fig. 1 ▸) which was isolated from NaNO_3_ through washing with water and filtration. Reagents were MnCl_2_·4H_2_O (Panreac, PRS), NaOH (Baker Analyzed) and NaNO_3_ (Merck Suprapur).

Elemental composition was determined from energy-dispersive X-ray (EDX) spectra obtained on a Philips XL30 scanning electron microscope (SEM) at 20 kV acceleration voltage, averaging data taken from three different homogeneous areas of ∼ 10 × 10 µm^2^, and on an EDS–ISIS Oxford spectrometer mounted on a Jeol 2010 TEM working at 200 kV, averaging data taken from nine areas on four different single rods.

Electron diffraction data collection was carried out using the EDT method (Kolb *et al.*, 2007 ▸, 2011 ▸). In EDT a series of patterns is collected while the crystal is tilted in steps around the goniometer axis. The reciprocal space falling between the recorded orientations is integrated by collecting the patterns in precession mode, *i.e.* the electron beam is precessed on a cone surface with the vertex fixed on the sample (Vincent & Midgley, 1994 ▸). The collected patterns are used to obtain a three-dimensional reconstruction of the investigated angular range of reciprocal space from which the unit-cell parameters and the extinction group can be derived. The combined effect of collecting patterns in random orientations and integrating the diffracted intensities over the excitation error makes the intensities extracted for these data sets close to the kinematical approximation and therefore suitable for structure solution (Mugnaioli *et al.*, 2009 ▸).

EDT data were collected on a Zeiss Libra 120 operating at 120 kV. The microscope is equipped with an in-column omega filter for energy-filtered imaging and a Nanomegas Digistar P1000 for precession electron diffraction. Data collection was performed by tilting the sample around the goniometer axis in an angular range of 110° (from −50 to +60°) in steps of 1°, and with a precession semiangle of 1°. The EDT patterns were energy filtered with a slit of 20 eV centred around the zero loss peak. It has been demonstrated that energy filtering is generally not strictly necessary for structure solution and refinement (Gemmi & Oleynikov, 2013 ▸; Palatinus, Corrêa *et al.*, 2015 ▸), but the patterns collected in this way show sharper peaks and a lower inelastic background.

The intensities were integrated using the PETS software (Palatinus, 2011 ▸). *Ab initio* structure determination was performed both by the direct methods implemented in *SIR*2011 (Burla *et al.*, 2012 ▸) and by charge flipping implemented in the *SUPERFLIP* software (Palatinus & Chapuis, 2007 ▸) embedded in *JANA*2006 (Petříček *et al.*, 2014 ▸). Refinement was performed both in a standard kinematical approach using *SHELX* (Sheldrick, 2008 ▸) and in the recently proposed dynamical approach of Palatinus *et al.* (2013 ▸), Palatinus, Corrêa *et al.* (2015 ▸) and Palatinus, Petříček & Corrêa (2015 ▸) included in *JANA*2006. For the dynamical refinement only 1 pattern out of 111 was excluded from the final calculation where the following parameters were used: 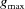 = 2 Å^−1^, 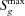 (matrix) = 0.01 Å^−1^, 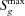 (refine) = 0.1 Å^−1^, 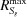 = 0.75, 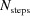 = 128. No geometrical restraint was imposed.

Laboratory X-ray diffraction patterns were obtained using a Panalytical X’pert powder diffractometer with Bragg–Brentano geometry, Ni-filtered Cu *K*α radiation (λ = 1.5405981 and 1.5444183 Å) and an X’Celerator linear position sensitive detector (more details in §1.1 and §2.1 of the supporting information).

A synchrotron X-ray diffraction pattern was obtained at the ID09 beamline at ESRF (Grenoble, France), using the standard beamline setup (Merlini & Hanfland, 2013 ▸), monochromatic radiation of λ = 0.415352 Å, glass capillary of 0.2 mm in diameter, beam diameter 0.8 mm, flat panel MAR555 detector at a distance of 300 mm, pixel size 139 × 139 µm. The X-ray powder pattern was collected during a full rotation of the sample and the two-dimensional powder rings were integrated into a conventional one-dimensional powder pattern using the *FIT2D* software (Hammersley, 1997 ▸), taking into account the geometrical and intensity corrections needed. High-quality diffraction data were obtained in the range 2θ = 1.309 to 32.245°, step size 0.012°, corresponding to a resolution of *d* = 18.2 to 0.748 Å.

The *GSAS* program system (Larson & Von Dreele, 2004 ▸, Version 2011dec ▸9 for Linux) combined with the EXPGUI graphical interface (Toby, 2001 ▸) was used for Rietveld refinement least-squares calculations. The background was simulated using a 15-term (up to 36 for synchrotron data) Chebyshev function, a correction of the pattern origin was allowed for, and peak profiles were calculated using a pseudo-Voigt function (Thompson *et al.*, 1987 ▸) providing for both instrument and material dependent parameters. The three instrument dependent profile parameters used (the Gaussian variances *U*, *V* and *W* of Caglioti *et al.*, 1958 ▸) were found from independent refinements using standard materials (3 µm silicon powder) and held constant throughout all calculations (laboratory data) or constrained to be equal for all phases (*W* in SR data).

## 3. Results   

### 3.1. Synthesis and composition   

The brown precipitate obtained after the first reaction of the two-step synthesis procedure, once dry, gives the diffraction pattern of hausmannite, a (possibly defective) spinel of composition Mn_3_O_4_ (or Mn_2_O_3_ = Mn_2.67_O_4_), whose structure is tetragonally distorted due to a Jahn–Teller effect in the 3*d* ^4^ electron configuration of Mn^3+^. The occurrence of Mn^3+^ indicates that, during the first reaction, manganese has been oxidized from 2 to 2.7 or 3.0. Subsequent calcination in NaNO_3_ gives the final product (Na_*x*_□_1 − *x*_)_5_[MnO_2_]_13_ whose composition corresponds, with *x* = 0.80, to an average oxidation state of 3.69 for manganese, *i.e.* oxidation must also accompany the second reaction, possibly through decomposition of nitrate NO_3_^−^ + *e* ^−^ → NO_2_ + O^2−^.

SEM images reveal that the sample consists of rods of < 4 µm in length and 30–60 nm in thickness (Fig. 1 ▸). Chemical composition was first determined using SEM-EDX on the loose powder samples, giving the ratio Na/Mn = 0.5 (2), with a high standard deviation due to sample rugosity and impurities. Some points, corresponding to denser masses in the SEM image, gave higher Na contents and might reflect Na_2_Mn_3_O_7_/birnessite impurities. Birnessite was also detected in the powder diffraction pattern (Fig. S1), and it cannot be excluded that particles of this compound are dispersed in the whole product and unavoidably sampled by the SEM-EDX probe (10 µm in diameter).

TEM-EDX was used to obtain chemical information from single rods. The analysis gave a ratio of Na/Mn = 0.22 (2) for nine points on four different crystals. This is probably more accurate, but values may tend to fall short due to Na evaporation during the electron bombardment, which is more important in TEM. Such evaporation could be observed from the fact that, at the beginning of some analyses, the Na peak at 1041 eV grew more rapidly than afterwards. It was anyway not possible to precisely quantify this effect. The best estimate is therefore the intermediate taken from structure refinement [Na/Mn = 0.306 (14)].

### 3.2. Crystal structure model from single-crystal electron diffraction intensities   

**Figure 2:**
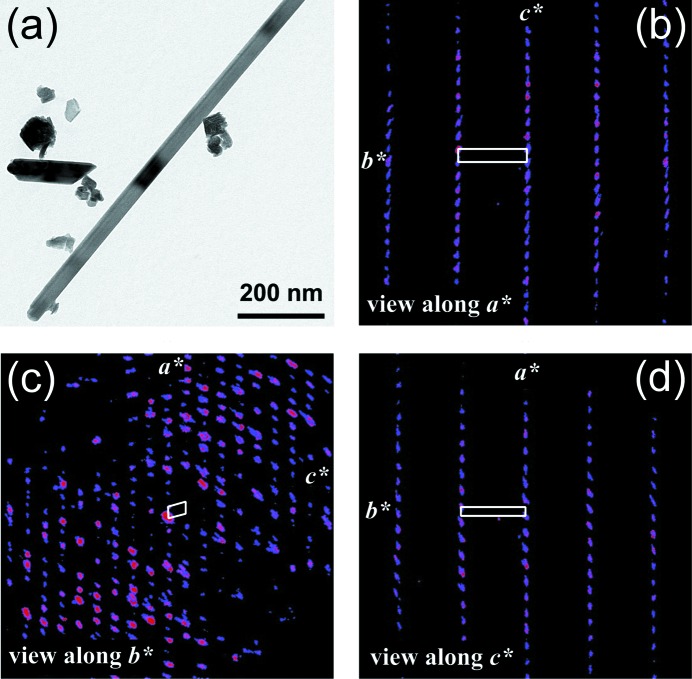
TEM image of the (Na,□)_5_[MnO_2_]_13_ rod selected for EDT data collection (*a*). Reconstructed EDT diffraction volume oriented along a* (*b*), b* (*c*) and c* (*d*). Note that (*b*, *c*, *d*) are projections of a three-dimensional volume and not conventional electron diffraction in-zone patterns. The projection of the reciprocal cell is sketched in white.

From EDT (Fig. 2 ▸), a *C*-centred monoclinic unit cell, *a* = 22.63 (12), *b* = 2.826 (14), *c* = 14.91 (7) Å, β = 104.6 (5)°, was unequivocally derived, the *a*, *c* and β parameters being very different from those in the romanèchite cell (*C*2/*m*, *a* = 13.929, *b* = 2.8459, *c* = 9.678 Å, β = 92.39°; Turner & Post, 1988 ▸). The main direction of growth of the rods is always **b**. The diffraction symbol is *2*/*mC*– – leaving *C*12/*m*1, *C*121 and *C*1*m*1 as possible space groups. *SUPERFLIP* gave space group *C*2/*m* as first choice for the correct solution. In order to obtain a confirmation about this space group, we conducted a supplementary statistical analysis of intensities using the program suite *DIFRASYM* (Gregorkiewitz & Vezzalini, 1989 ▸). A value of *pwys*(*h*0*l*) = 0.900 suggests that –1/*m*– is either absent or most atoms lie on the reflection plane (which is actually the case), and the intensity distribution parameters (Ramachandran & Srinivasan, 1959 ▸) *NYQ1*(*hkl*) = 0.456 and *NYQ1*(*h*0*l*) = 0.699 comply with the presence of the centre 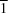 and the binary –2–, respectively (*NYQ1* = 1.960 for acentric and 0.776 for centric distribution). We therefore choose *C*2/*m* to start with model search and parameter refinement. The internal error for averaging over Laue equivalent intensities is *R*_sym_ = 0.135 and clearly within the mean error of all intensities *R*_σ_ = Σσ*I*/Σ*I* = 0.157 (Table S1).

**Figure 3:**
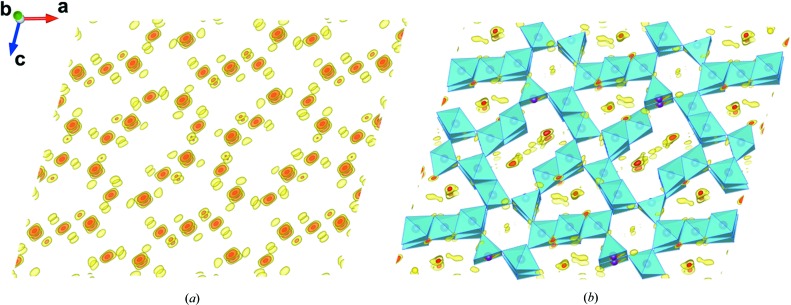
(*a*) Fourier synthesis calculated from the structure solution obtained by *SUPERFLIP* on the basis of EDT data, projected along a direction close to [010]. (*b*) Final framework model superimposed on a projection of the difference-Fourier map calculated with structure factors from EDT intensities and phases from the framework only.

In the structure solutions obtained both with *SUPERFLIP* and *SIR*2011 ▸ we recognized a preliminary model which contained all framework atoms (7 Mn and 13 O sites). In addition, as for other tunneled structures solved by EDT data (Rozhdestvenskaya *et al.*, 2010 ▸), electron densities in the channels showed up in a difference Fourier map and were assigned, in this case, to different Na sites. In Fig. 3 ▸ we report the reconstructed electron density, given by the *SUPERFLIP* solution in which the framework topology is evident, and the difference Fourier map superimposed to the final structure model, where two main Na sites, one inside the S-shaped 10-ring channel and the other in the 8-ring channel, are clearly visible along with some weaker residuals in the channels.

### 3.3. Structure refinement   

The so-obtained structure was subsequently refined by the Rietveld method. The first trial, using laboratory X-ray powder diffraction data, confirmed the EDT overall model providing for improved unit-cell parameters, but convergence was achieved only after the Mn—O distances were restrained using the distance least squares (DLS) method (Meier & Villiger, 1969 ▸) and no improved structural parameters could be obtained, probably due to a problem with peak resolution (see §S4.1).

We therefore substituted the laboratory pattern with a synchrotron radiation (SR) powder diffraction pattern. Their detailed inspection (Fig. S1) shows that in the SR pattern the reflection width is reduced by a factor of ∼ 3 (the *FWHM* of reflection 602 passes from 0.14° to 0.042° 2θ), but resolution in terms of (∂(2θ)/∂*NR*)/*FWHM* remains approximately the same due to the much shorter SR wavelength. However, the total number of peaks is halved (no α_2_ component), and a huge improvement of the signal-to-noise ratio can be seen, especially at high angles (Figs. S1 and 4 ▸).

With these improvements Rietveld refinement converged rapidly. For the final model, presented in Fig. 5 ▸ as well as in Table S2 and the CIF file in the supporting information, refinement included several parameters of the impurity phases birnessite (those specified in Table 1 ▸ plus eight atom parameters) and Na_2_Mn_3_O_7_ (unit cell and Lorentzian broadening only). Attempts to refine anisotropic grain shape, microstrain (Stephens, 1999 ▸) and preferred orientation (ODF) were made and showed that these phenomena have little relevance. A final agreement of χ^2^ = 0.690, *R*_wp_ = 0.051, *R*_p_ = 0.037, *R*_*F*2_ = 0.035 was reached (Table 1 ▸). With respect to the refinement using the laboratory X-ray pattern, the structural agreement for the (Na,□)_5_[MnO_2_]_13_ phase alone, *R*_*F*2_ = 0.036 instead of 0.10, has greatly improved. The model, corroborated by extensive significance tests in the final stage of refinement (see §S4.2 and S7.1), clearly shows that Na occupies all three channels while Mn—O distances in the framework, now free from restraints, diversify to comply with an ordered Mn^3+^–Mn^4+^ distribution. These details are fundamental to the chemical behaviour and will be discussed later.

**Figure 4:**
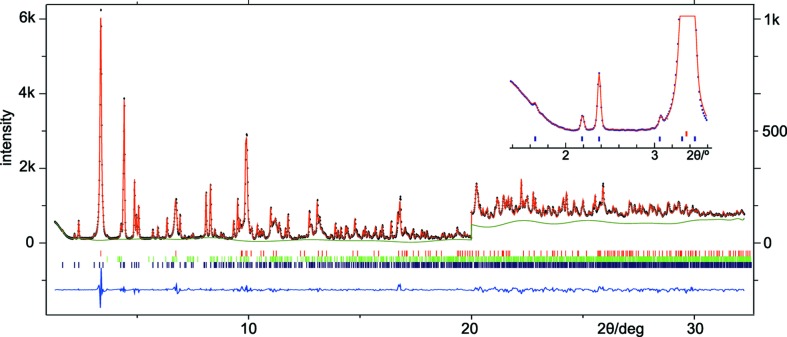
Observed (*Y*_o_, dots), calculated (*Y*_c_, line), difference (*Y*_o_ − *Y*_c_, below) and background (*Y*_b_, smooth line) intensities as obtained after Rietveld refinement using synchrotron data. All intensities are multiplied by 6 for 2θ ≥ 20° to show details. Ticks give reflection positions, from top to bottom, for birnessite, Na_2_Mn_3_O_7_ and (Na,□)_5_[MnO_2_]_13_. The inset shows the fit in the low-angle region which is important for Na site occupation factors (see text). λ = 0.415352 Å.

The excellent agreement between observed and calculated intensities can also be judged from the patterns in Fig. 4 ▸ where all discrepancies with |*Y*_o_ − *Y*_c_|/σ*Y* > 3 lie in regions of important birnessite peaks, *i.e.* they are due to errors in the model used to describe the birnessite and not the (Na,□)_5_[MnO_2_]_13_ structure. Details about the modeling of birnessite are interesting in their own right and suggest (see §S4.2) that this typically hydrothermal phase, not expected in our salt melt synthesis, was derived from Na_2_Mn_3_O_7_, a layered structure which forms at high temperatures (Chang & Jansen, 1985 ▸; Raekelboom *et al.*, 2001 ▸) and may then hydrate (Parant *et al.*, 1971 ▸; Chen *et al.*, 1996 ▸; Caballero *et al.*, 2002 ▸; Nam *et al.*, 2015 ▸), during the washing procedure when isolating the product from NaNO_3_.

In order to further confirm the details of the structural model for (Na,□)_5_[MnO_2_]_13_, we subsequently undertook several refinements using single-crystal electron diffraction intensities. A comparison with the results from powder diffraction also gives the opportunity to check if the bulk structure corresponds to the model obtained from a single crystal a few hundred nanometres in size.

In a first approach, the raw model was input to a regular single-crystal structure refinement through least squares and Fourier cycles using the kinematical approximation by the program *SHELX*97 (Sheldrick, 2008 ▸). Refinement was stable and converged rapidly, without imposing any geometrical restraint, to *R*1(*F*) = 0.263 (Table S1), but the framework geometry still showed some dispersion (*cf.* Table S4) and, among the three sites for Na, only the two in the 10- and the 8-ring channels were resolved.

In a second trial, refinement was continued using the recently developed method based on dynamical diffraction theory (Palatinus *et al.*, 2013 ▸; Palatinus, Petříček & Corrêa, 2015 ▸). Convergence was now reached at a residual of *R*(*F*) = 0.07 (0.24) for observed (all) intensities, and the resulting model (Table S2 and the CIF file in the supporting information) contains all atoms, including individual atomic displacement parameters, and atom parameters are near to those obtained from Rietveld refinement. These results are remarkably reliable for a structure derived from electron diffraction intensities, especially when compared with the model derived by kinematical theory. Details about structural features and related uncertainties will be discussed later.

## 4. Discussion   

### 4.1. Charge ordering and Na coordination   

An inspection of the Mn—O distances (Table 2 ▸) clearly indicates an ordered distribution of Mn^3+^ and Mn^4+^ over the seven available sites. A composition (Na_*x*_□_1 − *x*_)_5_[MnO_2_]_13_ with *x* = 0.80 requires that 4/13 Mn atoms occur as Mn^3+^. From interatomic distances, the corresponding sites are Mn4, in an octahedron with 〈*d*(Mn—O)〉 = 2.01 Å, and Mn7, in a square pyramid with 〈*d*(Mn—O)〉 = 1.95 Å. Both distances come close to the values calculated from ionic radii [2.005 Å for high-spin Mn^3+^(VI) and 1.94 Å for Mn^3+^(V), respectively; Shannon, 1976 ▸] and are well distinguished from those of the Mn1, Mn3, Mn5 and Mn6 sites which range from 1.88 to 1.92 Å and correspond to Mn^4+^(VI) (1.890 Å; Shannon, 1976 ▸). Mn2 is intermediate with 〈*d*(Mn—O)〉 = 1.94 Å.

In addition, a pronounced Jahn–Teller distortion, expected for high-spin 3*d* ^4^ electron configuration, can be recognized for both Mn4 and Mn7. In Mn4, the longer distances are found on the O6—Mn—O8 axis (2.150 and 2.037 Å, in the ***ca*** plane) defining the filled *e*_*g*_ orbital, and Mn7 lies far away from the vertex [*d*(Mn7—O3) = 2.172 Å], practically on the basis of the square pyramid (2 O5 + 2 O12). According to the model refined on the basis of EDT data and dynamical theory, Mn2 also presents a (less pronounced) Jahn–Teller distortion, that was not evident in the PXRD Rietveld refined model (Tables 2 ▸ and S3).

At a first glance, the coordination number *CN* = 5 of Mn7 might be surprising, but square pyramids for Mn^3+^ are also found, *e.g.* in the sheet structure Na_4_Mn_2_O_5_ (Brachtel & Hoppe, 1980 ▸) and in the tunnel structure Na_4_Mn_9_O_18_ (*e.g.* Chu *et al.*, 2011 ▸), with the Mn—O distances 1.953 × 2, 1.941 × 2, 2.068 Å and 1.892 × 2, 1.926 × 2, 2.146 Å, respectively.

In order to establish the *CN* of sodium, distances were calculated up to 4 Å and a clear gap between the first [*d*(Na—O) < 2.9 Å] and the second [*d*(Na—O) > 3.1 Å] coordination shell was found. Distances in the first shell are reported in Table 2 ▸ and give the canonical coordination environment of sodium with *CN* = 7, 8 and 6 for Na1, Na2 and Na3, respectively. For the dynamics of the structure it is important to realise that Na1 and Na2 stay in a trigonal prism with one (Na1) or two (Na2) more oxygen ligands on their faces, which provides reasonable electrostatic shielding. Na3, on the other hand, stays at the centre of a trigonal antiprism, extremely flattened along **b**, which lacks shielding along the tunnel axis. The alternative Na3 position at *y* = 0, mentioned in the results section §S4.2, has *CN* = 9 with highly dispersed Na—O distances ranging from 2.07 to 2.85 Å, *i.e.* neither of the two positions provides a suitable environment for Na^+^ and it is possible that, as a consequence, Na3 may move more easily along the channel.

### 4.2. A new framework: tunnels and possible transformations   

**Figure 5:**
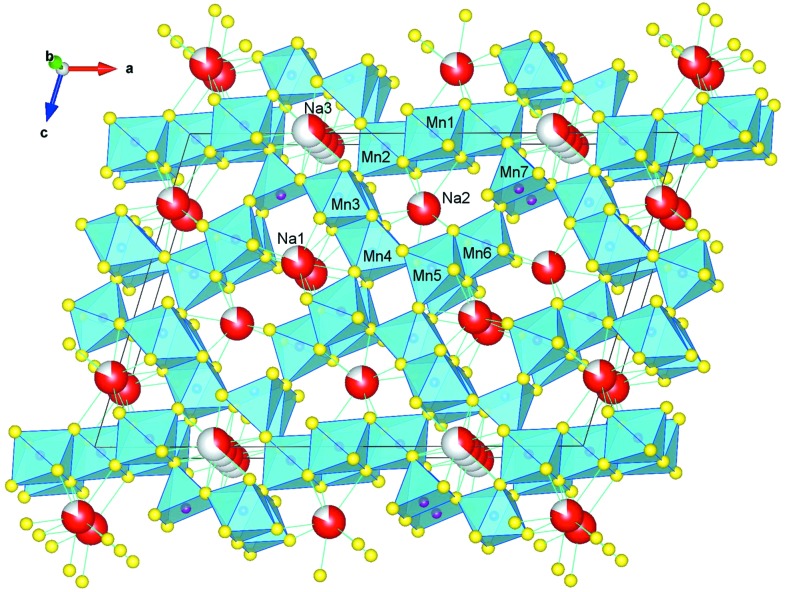
Crystal structure of (Na_*x*_□_1 − *x*_)_5_[MnO_2_]_13_, *x* = 0.80. The [MnO_2_] framework is built up by MnO_6_ and MnO_5_ polyhedra (sky-blue) leaving three types of channels along **b**, two large S-shaped channels each containing < 2 Na, four egg-shaped channels containing < 1 Na each, and two small six-ring channels which contain again < 1 Na each (split on 2 positions). Image created using *VESTA* (Momma & Izumi, 2011 ▸).

The framework of (Na,□)_5_[MnO_2_]_13_ (Fig. 5 ▸) is very different from that of romanèchite {2 × 3 tunnel structure, chemical formula (Ba,H_2_O)_2_[MnO_2_]_5_} and resembles the one first found by Mumme (1968 ▸) for Na_4_[Mn_4_Ti_5_O_18_] and later refined (Richardson *et al.*, 1998 ▸; Akimoto *et al.*, 2011 ▸; Chu *et al.*, 2011 ▸; Kruk *et al.*, 2011 ▸) for Na_3.6–4.5_Mn_9_O_18_ = Na_0.4–0.5_[MnO_2_]. In both structures there are tunnels, running along the short (2.8 Å) axis, which are defined by walls of double and triple chains of octahedra, occasionally replaced by a single chain made up of square pyramid Mn^V^O_5_ polyhedra. The most visible difference among the two frameworks is that the Mumme (1968 ▸) structure is reminiscent of ramsdellite with its 1 × 2 tunnels, while our compound recalls the 2 × 2 tunnels of hollandite.

**Figure 6:**
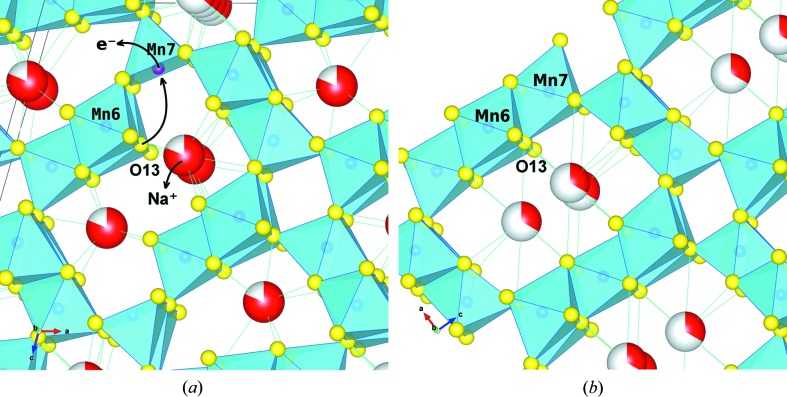
Detail of the crystal structure of (Na,□)_5_[MnO_2_]_13_, showing the possible topotactic transition of an S-shaped tunnel (*a*) to a 2 × 3 romanèchite tunnel (*b*). Oxidation of the framework NaMn^3+^ → □Mn^4+^ induces the nucleophilic attack of O13 at Mn7.

In the classical tunnel structures (Pasero, 2005 ▸), to which romanèchite belongs, all Mn atoms are octahedrally coordinated and all O atoms are shared by three octahedra, thus defining the framework stoichiometry MnO_6/3_ = MnO_2_. In (Na,□)_5_[MnO_2_]_13_, Mn7 lacks one oxygen and becomes MnO_5/3_, which is compensated by Mn6 where the two O13 O atoms (Fig. 6 ▸) are shared by only two octahedra giving MnO_4/3_O_2/2_ = MnO_7/3_.

The existence of such disproportionations suggests possible pathways in the synthesis of OMS frameworks. Solid-state transformations from a birnessite layer structure to one of the different tunnel structures are presently much discussed (Drits *et al.*, 1997 ▸; Lanson *et al.*, 2002 ▸; Li & Wu, 2009 ▸; Grangeon *et al.*, 2014 ▸). Here it can be seen that a five-coordinated Mn7, on oxidation from Mn^3+^ to Mn^4+^, may become the target for nucleophilic attack, *e.g.* by O13 which is nearest among second coordination sphere O atoms (2.88 Å) and, through a bond to Mn7, would reach the sharing coefficient 3 adopted by all other O atoms in (Na,□)_5_[MnO_2_]_13_ and usually found in the tunnel structures. If this happened systematically, the S-shaped tunnel would transform into the 2 × 3 romanèchite tunnel (Fig. 6 ▸). Independent support for such speculations comes from a recent DFT study on alkali hollandites (Tompsett & Islam, 2013 ▸), where a progressive increase of the in-plane Mn—O distances was seen to accompany reduction.

The coupling between redox and topotactic transformation mechanisms is important not only for synthesis but also for electrochemical applications of manganese oxide materials. Much of the limits in *x* for the (de)intercalation reaction [MnO_2_] + *x* *M* ^+^ + *xe* ^−^ ↔ *M*_*x*_[MnO_2_] are indeed due to structural transformations that compromise reversibility (see *e.g.* the discussion of deep discharge in Hu & Doeff, 2004 ▸).

### 4.3. Chemical formula and preferred compositions   

(Na,□)_5_[MnO_2_]_13_ has three different channels which are only partially filled with Na, evidently also a consequence of the relatively short period along *b* = 2.84 Å which implies strong repulsive Na^+^—Na^+^ interactions in the Na chains along **b** (comparatively, the lateral distance between the adjacent Na1 chains in the S-shaped tunnel is 3.94 Å). From structure refinement, we find an average degree of filling of 0.8 in all Na chains and, in principle, there might be some multiple or incommensurate period to accommodate sodium in an orderly way. However, inspection of overexposed electron diffraction patterns showed only a very weak diffuseness along 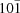 and no satellite peaks, suggesting an essentially statistical Na distribution. Dynamical refinement on the basis of EDT data allowed anisotropic displacement parameters to be introduced for all metal atoms except Na3 and it turned out that Na atoms have a relatively larger *U* ^22^ component compared with Mn atoms. This supports the idea of a certain disordered distribution of cations along the channels (see Table S2).

The actual number of Na per unit cell is 8, even if there is place for 10 Na (see Table S2). The exact match of 8 Na with 2 × 4 = 8 Mn^3+^ positions suggests that charging of the framework, *e.g.* in a redox reaction during synthesis or in electrochemical cycling, is not a fully statistical process but follows a stepwise reduction of different Mn sites, so we expect pronounced voltage/composition plateaus.

The highest charge, corresponding to a load of 10 Na per unit cell, corresponds to the ratio Na/Mn = 10/26 = 0.385, near to the composition Na_0.40_[MnO_2_] first suggested by Parant *et al.* (1971 ▸). There are few analytical data. Tsuda *et al.* (2003 ▸) give Na/Mn = 0.31 for a product calcined at 873 K in air, exactly the same value as found for our material, and (Li + Na)/Mn = 0.38 for an ion-exchanged derivative (LiNO_3_, 623 K, under Ar). Hu & Doeff (2004 ▸) found instead Na/Mn = 0.41 for calcination at 873 K in the presence of an organic reducing agent, and (Li + Na)/Mn = 0.33 and 0.40 for the ion-exchanged derivatives (LiBr in EtOH, 353 K, air, and LiNO_3_/LiNO_2_, 473 K, air, respectively). It would be interesting to check the structure of these materials: if the framework of (Na,□)_5_[MnO_2_]_13_ is conserved, as the published X-ray diffraction patterns suggest, we might have a transition between structures with *x* = 0.80 and *x* = 1.00 as predicted from our chemical formula. Correspondingly, a further 2 Mn per unit cell must undergo reduction to Mn^3+^, possibly at Mn2 which has the longest mean Mn—O distance [*d*(Mn—O) ≥ 1.94 Å, Table 2 ▸] after Mn4 and Mn7, but a rearrangement of charges cannot be excluded.

Regarding the lowest sodium content, both Tsuda *et al.* (2003 ▸) and Hu & Doeff (2004 ▸) conclude, from electrochemical measurements, that higher oxidation states of the framework (down to *x* = 0.07) should also exist. Our results suggest that each oxidation state should comply with an ordered Mn^3+^—Mn^4+^ distribution, *i.e.* we expect a preference for *x* = 0, 0.15, 0.31 and 0.39, in excellent agreement with the results from chemical analysis and electrochemical measurements.

Interestingly, and in contrast with our material, the Mumme (1968 ▸) framework cannot be fully oxidized and always retains Mn^3+^ in the square pyramid (Mn4 site, see §S6.1). This may be a consequence of the different environments of the square pyramids: on oxidation, in our case the square pyramid can easily convert to an octahedron through incorporation of O13 (Fig. 6 ▸), whereas in the Mumme (1968 ▸) framework (*cf.* Fig. 1 ▸ in Doeff *et al.*, 2004 ▸) there are no ‘under-shared’ O atoms (like O13 in Fig. 6 ▸) at reach and a similar mechanism would be hard to explain. This reduces the theoretical capacity from 182 mAh g^−1^ to (8/12) × 182 = 121 mAh g^−1^, coming near to the capacity of (Na,□)_5_[MnO_2_]_13_ which is 108 mAh g^−1^ (calculated from structure) and ∼ 90 mAh g^−1^ (measured from electrochemical cycling; Tsuda *et al.*, 2003 ▸; Hu & Doeff, 2004 ▸).

### 4.4. Contrasting with the romanèchite framework   

Knowledge about synthetic materials with the ‘true’ romanèchite framework (2 × 3 tunnels, right part of Fig. 6 ▸) is limited. Tsuda *et al.* (2001 ▸) synthesized (453 K, autogenous pressure) an analog to the natural material, with composition Ba_0.18_MnO_2.10_·0.42H_2_O, and studied its performance as a positive electrode in a Li cell. The charge–discharge curves from 2 to 4 V are almost featureless, without the intermediate plateaus observed by the same authors (Tsuda *et al.*, 2003 ▸) for (Na,□)_5_[MnO_2_]_13_. The corresponding material is actually a mixture between barian and lithian compositions and interpretations must be done with caution, but in principle the romanèchite structure (*M*,H_2_O)_2_[MnO_2_]_5_ possesses only one crystallographically distinct site for the tunnel cation *M* or water and would be in agreement with a monotonous charge–discharge curve.

Later, Shen *et al.* (2004 ▸) reported the synthesis of a sodian romanèchite with composition (Na_0.24_(H_2_O)_0.16_)[MnO_2_]·0.55H_2_O. Again, the material was obtained from hydrothermal synthesis (∼ 493 K, autoclave), and from thermal analysis it was concluded that water occupies part of the tunnel sites where it has also been found for the mineral (Wadsley, 1953 ▸; Turner & Post, 1988 ▸).

The structural identity of the above two materials was inferred (Tsuda *et al.*, 2001 ▸; Shen *et al.*, 2004 ▸) from their powder X-ray diffraction patterns which resemble the reference pattern for natural romanèchite (PDF Powder Diffraction File, Card #14-627, JCPDS – International Centre for Diffraction Data^®^, 12 Campus Blvd, Newtown Square, PA 19073-3273 USA, 1997–2015). Neither of the two patterns has been indexed, but for the sodian material, the typical unit-cell dimensions of romanèchite were confirmed from high-resolution transmission electron micrographs. Also, the pattern of the sodian material (Fig. 1 in Shen *et al.*, 2004 ▸) grossly differs from our pattern (Fig. S1), especially for the all important low-angle peaks. A romanèchite proper material therefore appears to be well distinguished from our (Na,□)_5_[MnO_2_]_13_ by both structure and formation conditions.

Finally, we may compare romanèchite, (*M*,H_2_O)_2_[MnO_2_]_5_, and the new structure, (Na,□)_5_[MnO_2_]_13_, in terms of their chemical formula and unit cell. While stoichiometry and cell dimensions are clearly different, their cavity/Mn ratio is almost identical (0.400 and 0.385) inviting considerable confusion since the day when Parant *et al.* (1971 ▸) first discovered the Na_0.40_[MnO_2_] material. Directly related, also the specific capacities are very similar (112 and 108 mAh g^−1^, respectively; values refer to the fully Na-loaded compositions). A striking difference can be seen, however, regarding the openness of their frameworks. In terms of framework densities *n*_Mn_ (number of Mn polyhedra per 1 nm^3^) we calculate *n*_Mn_ = 28.3, 26.7 and 26.1 nm^−3^ for the (Na,□)_5_[MnO_2_]_13_, Mumme (1968 ▸) and romanèchite frameworks, which can be compared with 35.8, 28.6 and 22.6 nm^−3^ for pyrolusite MnO_2_, hollandite (*M*,□)[MnO_2_]_4_ and todorokite Mg(H_2_O,*M*,□)_4_[MnO_2_]_6_, three well known representatives of OMS with 1 × 1, 2 × 2 and 3 × 3 tunnels. In this series, romanèchite is seen to be considerably more open than (Na,□)_5_[MnO_2_]_13_, and different kinetical properties can be expected.

### 4.5. Reliability of results from EDT single-crystal and X-ray powder diffraction   

The structure of (Na,□)_5_[MnO_2_]_13_ was finally revealed combining EDT based *ab* *initio* structure model determination and Rietveld PXRD structure refinement. As excellently pointed out by McCusker & Baerlocher (2009 ▸), electron diffraction and PXRD are rather complementary methods, whose combination may be extremely powerful for the structure investigation of nanocrystalline materials. Crucial steps forward for electron diffraction derived from the development of beam precession (Vincent & Midgley, 1994 ▸) and tomographic methods for data collection and analysis (Kolb *et al.*, 2007 ▸; Mugnaioli *et al.*, 2009 ▸; Zhang *et al.*, 2010 ▸) which made it possible to acquire more complete and more kinematical electron diffraction data sets, are able alone to deliver *ab* *initio* a first structure model that can be subsequently refined by Rietveld methods. This strategy has proved successful for the characterization of tetrahedral molecular sieves (Jiang *et al.*, 2011 ▸; Bellussi *et al.*, 2012 ▸; Martínez-Franco *et al.*, 2013 ▸).

In the present case, we were also able to perform a single-crystal refinement on the basis of EDT intensities using the dynamical refinement method recently developed by Palatinus *et al.* (2013 ▸; Palatinus, Petříček & Corrêa, 2015 ▸). This is one of the first cases where this new approach was applied for the refinement of an unknown structure, giving us the opportunity to compare between results obtained from different data and methods (details in §S7.1).

Atom positions obtained *ab* *initio* (by a kinematical approach) on the basis of EDT data already embodied a reasonably correct model for the MnO_2_ octahedral framework of (Na,□)_5_[MnO_2_]_13_, despite the structure residual of about *R*1(*F*) = 0.263 (Table S1). Mn and O atom positions could be straightforwardly assigned and were stable after least-squares refinement without imposing any restraint or constraint. Most of the Na positions could be deduced from the difference Fourier map, even if their occupancy and displacement factor could not be refined.

Rietveld refinement using laboratory X-ray powder data served, in our case, for a first improvement of the unit cell (where EDT gives uncertainties on the order of 0.5%) and to check the correctness of the EDT model which, after introducing DLS restraints to avoid correlations, refined to a theory-biased rough model (*R*_*F*2_ = 0.10) where Mn—O distances scatter tightly around the imposed mean [〈MnO〉 = 1.89 (2) Å].

SR powder data, beyond a further improvement of the unit-cell parameters (uncertainties are now less than 5 × 10^−5^, Table 1 ▸), allowed unrestrained Rietveld refinement and gave details like the ordered Mn^3+^—Mn^4+^ distribution and the Na3 site occupation factor which was important to fit the intensities of the low-angle peaks (see Fig. 4 ▸).

EDT data combined with dynamical scattering refinement essentially confirm the model obtained from SR data. By using the Bilbao Crystallographic Server (Tasci *et al.*, 2012 ▸), the average (maximum) discrepancies between the two coordinate sets were found to be 8 (21) pm for all and 3 (5) pm for the Mn atoms. This is ∼ 5 (10) times the uncertainty estimated from least-squares calculations with SR(EDT) data, and ∼ 4 times the discrepancies reported in test runs for dynamical scattering refinements (Palatinus, Corrêa *et al.*, 2015 ▸). For future work it will be interesting to explore the significance of these discrepancies.

Here we are mainly concerned with the structural results, and their detailed inspection (§S7.1) shows that differences do not affect the interpretations put forward in the preceding sections, *i.e.* the similarity of two independent results can be taken as an additional warranty of their correctness. One discrepancy which should be highlighted regards the Mn2 octahedron. While both SR data Rietveld refinement and EDT dynamical refinement give very much the same mean 〈Mn—O〉 distances [1.94 (8) and 1.94 (5) Å, respectively], complying with some Mn^3+^ substitution, only the latter shows clearly the expected Jahn–Teller distortion with the long axis (O2—Mn2—O4) in the ***ca*** plane.

Finally, we point out that EDT dynamical refinement allowed to refine all structure parameters without any constraint, including displacement parameters for all atoms, up to very reasonable values. For all Mn and two out of three Na atoms it was also possible to refine anisotropic displacement parameters, showing that for all Mn atoms *U* ^22^ is systematically smaller than *U* ^11^ and *U* ^33^ and that, conversely, for at least one Na atom *U* ^22^ (parallel to the channel direction) is larger.

## 5. Conclusions   

(Na_*x*_□_1 − *x*_)_5_[MnO_2_]_13_ was synthesized using a new and facile procedure which yielded nanorods with the Na load *x* = 0.80. The long-awaited crystal structure of this material has been resolved and shows a novel OMS framework containing three distinct types of tunnel, which differs radically from the previously assumed romanèchite framework containing only one type of tunnel. A particularly interesting detail of the new framework is the existence of MnO_5_ square pyramids which, on oxidation from Mn^3+^ to Mn^4+^, may act as centres for nucleophilic attack from a nearby under-shared oxygen. This mechanism is likely to play a fundamental role for both synthesis and electrochemical behaviour of manganese-based OMS structures.

The elucidation of this particular and quite complex structure has become possible through EDT-based *ab* *initio* model determination combined with SR powder diffraction based Rietveld refinement. The procedure was straightforward and led rapidly to a model whose precision (positional errors < 1.5 pm) can be compared with ordinary single-crystal refinement except for atomic displacement parameters. This opens new opportunities for the development of OMS materials where progress is often difficult due to their cryptocrystalline and polyphasic nature.

As a novelty for an unknown structure, a single-crystal refinement based on EDT data and dynamical scattering theory has been performed and it could be shown that results compete in precision with those obtained from SR data and can be taken to confirm the reliability of the final model.

## 6. Related literature   

References cited in the supporting information include: Armstrong *et al.* (1998 ▸), David (2001 ▸), Drits *et al.* (2007 ▸), Jeong & Manthiram (2001 ▸), Kim *et al.* (2012 ▸), Sauvage *et al.* (2007 ▸), Tian & Billinge (2011 ▸).

## Supplementary Material

Crystal structure: contains datablock(s) ROMAN7_.25_publ, ROMANE_dyn_aniso. DOI: 10.1107/S2052520616015651/dk5050sup1.cif

Structure factors: contains datablock(s) ROMAN7_.25_publ. DOI: 10.1107/S2052520616015651/dk5050ROMAN7_.25_publsup2.hkl

Rietveld powder data: contains datablock(s) ROMAN7_.25_publ. DOI: 10.1107/S2052520616015651/dk5050ROMAN7_.25_publsup3.rtv

Structure factors: contains datablock(s) I. DOI: 10.1107/S2052520616015651/dk5050ROMANE_dyn_anisosup4.hkl

Supporting information. DOI: 10.1107/S2052520616015651/dk5050sup5.pdf

CCDC references: 1508200, 1508201

## Figures and Tables

**Table 1:** Crystal data and overall parameters obtained from Rietveld refinement using SR data The title compound is (Na_*x*_□_1 − *x*_)_5_[MnO_2_]_13_, *x* = 0.80 (4), space group *C*2/*m*, *Z* = 2; for refined atom parameters see Table S1. Atom parameters of impurities were taken from Post & Veblen (1990 ▸) for birnessite and Raekelboom *et al.* (2001 ▸) for Na_2_Mn_3_O_7_. Estimated standard deviations (in parentheses) refer to the last digits of the preceding value. Linear absorption coefficient μ for λ = 0.415352 Å calculated from Henke *et al.* (1993 ▸). *NY*, *NR* and *NP* give the number of observations, reflections and refined parameters, respectively.

Parameter	Global	Title compound	Birnessite	Na_2_Mn_3_O_7_
*a* (Å)		22.5199 (6)	4.951 (2)	6.636 (3)
*b* (Å)		2.83987 (6)	2.8539 (9)	6.825 (3)
*c* (Å)		14.8815 (4)	7.2910 (10)	7.557 (4)
α (°)		90	90	105.43 (4)
β (°)		105.0925 (16)	104.13 (3)	107.62 (6)
γ (°)		90	90	111.43 (4)
*V* (Å^3^)		918.90 (4)	99.90 (5)	274.9 (3)
*MM*_uc_ (g mol^−1^)		2443.56	197–216†	645.574
ρ_calc_ (g cm^−3^)		4.416	3.3–3.6†	3.899
μ (cm^−1^)		18.5		
Mass fraction	1	0.579 (2)	0.401 (3)	0.020 (1)
*LX* (cdeg)		1.21 (6)	2.1 (2)	2.9 (5)
*LY* (cdeg^2^)		11.0 (5)	153 (4)	8 (9)
*NY*	2579	–	–	–
*NR*	2897	1374	151	1372
*NP*	123	61	15	9
χ^2^	0.690	–	–	–
*R*_wp_	0.051	–	–	–
*R*_p_	0.037	–	–	–
*R*_*F*2_	0.035	0.036	0.018	0.043

† Exact values depend on Mn vacancies and the interlayer Na/H_2_O ratio, not studied here.

**Table 2:** Interatomic distances (Å) for (Na_*x*_□_1 − *x*_)_5_[MnO_2_]_13_, *x* = 0.80 (4) as obtained from Rietveld refinement using SR data (the corresponding values obtained from dynamical refinement can be found in Table S3) Figures in parentheses refer to the last digits and are the standard deviations obtained from least-squares refinement for individual distances, and dispersions obtained from averaging over one or more polyhedra for mean distances. For global means, Mn4 and Mn7 are considered as Mn^3+^.

Mn1—O1 ×2	1.901 (17)	Mn2—O1 ×2	2.041 (12)	Mn3—O4 ×2	1.923 (14)
Mn1—O2 ×4	1.911 (10)	Mn2—O2	1.940 (17)	Mn3—O5	1.947 (16)
		Mn2—O3 ×2	1.871 (10)	Mn3—O6 ×2	1.936 (12)
		Mn2—O4	1.872 (20)	Mn3—O7	1.783 (15)
Mean	1.908 (5)	Mean	1.94 (8)	Mean	1.91 (6)
					
Mn4—O6	2.150 (14)	Mn5—O8 ×2	1.869 (10)	Mn6—O10	1.870 (16)
Mn4—O7 ×2	2.041 (13)	Mn5—O9	1.897 (17)	Mn6—O11 ×2	1.924 (11)
Mn4—O8	2.037 (15)	Mn5—O10 ×2	1.962 (11)	Mn6—O12	1.849 (16)
Mn4—O9 ×2	1.887 (12)	Mn5—O11	1.944 (17)	Mn6—O13 ×2	1.868 (11)
Mean	2.01 (10)	Mean	1.92 (4)	Mean	1.88 (3)
					
Mn7—O3	2.172 (16)		30×	〈Mn^4+^—O〉	1.91 (5)
Mn7—O5 ×2	1.904 (11)		11×	〈Mn^3+^—O〉	1.98 (11)
Mn7—O12 ×2	1.893 (11)		41×	〈〈Mn—O〉〉	1.93 (8)
Mean	1.95 (12)				
					
Na1—O6 ×2	2.587 (19)	Na2—O1 ×2	2.475 (16)	Na3—O3 ×2	2.385 (15)
Na1—O9	2.656 (24)	Na2—O2	2.879 (25)	Na3—O4 ×2	2.652 (16)
Na1—O10 ×2	2.510 (18)	Na2—O7 ×2	2.584 (18)	Na3—O5 ×2	2.276 (15)
Na1—O13 ×2	2.368 (17)	Na2—O8	2.472 (22)		
		Na2—O11 ×2	2.553 (18)		
Mean	2.51 (11)	Mean	2.57 (13)	Mean	2.44 (17)

## References

[bb1] Akimoto, J., Hayakawa, H., Kijima, N., Awaka, J. & Funabiki, F. (2011). *Solid State Phenom.* **170**, 198–202.

[bb76] Armstrong, A. R., Huang, H., Jennings, R. A. & Bruce, P. G. (1998). *J. Mater. Chem.* **8**, 255–259.

[bb2] Bellussi, G., Montanari, E., Di Paola, E., Millini, R., Carati, A., Rizzo, C., O’Neil Parker, W. Jr, Gemmi, M., Mugnaioli, E., Kolb, U. & Zanardi, S. (2012). *Angew. Chem. Int. Ed.* **51**, 666–669. 10.1002/anie.201105496 22131222

[bb3] Brachtel, G. & Hoppe, R. (1980). *Z. Anorg. Allg. Chem.* **468**, 130–136.

[bb4] Breck, D. W. (1974). *Zeolite Molecular Sieves: Structure, Chemistry, and Use.* Chichester, UK: Wiley and Sons.

[bb5] Burla, M. C., Caliandro, R., Camalli, M., Carrozzini, B., Cascarano, G. L., Giacovazzo, C., Mallamo, M., Mazzone, A., Polidori, G. & Spagna, R. (2012). *J. Appl. Cryst.* **45**, 357–361.

[bb6] Caballero, A., Hernán, L., Morales, J., Sánchez, L., Santos Peña, J. & Aranda, M. A. G. (2002). *J. Mater. Chem.* **12**, 1142–1147.

[bb7] Caglioti, G., Paoletti, A. & Ricci, F. P. (1958). *Nucl. Instrum.* **3**, 223–228.

[bb8] Camblor, M. A. & Hong, S. B. (2010). *Porous Materials*, edited by D. W. Bruce, D. O’Hare and R. I. Walton, pp. 263–325. New York: Wiley.

[bb9] Chang, F. M. & Jansen, M. (1985). *Z. Anorg. Allg. Chem.* **531**, 177–182.

[bb10] Chen, R., Chirayil, T., Zavalij, P. & Whittingham, M. S. (1996). *Solid State Ionics*, **86–88**, 1–7.

[bb11] Chu, Q., Wang, X., Li, Q. & Liu, X. (2011). *Acta Cryst.* C**67**, i10–i12. 10.1107/S0108270110052856 21285490

[bb12] Cundy, C. S. & Cox, P. A. (2003). *Chem. Rev.* **103**, 663–702. 10.1021/cr020060i 12630849

[bb77] David, W. I. F. (2001). *J. Appl. Cryst.* **34**, 691–698.

[bb13] Doeff, M. M. (1996). *J. Electrochem. Soc.* **143**, 2507–2516.

[bb14] Doeff, M. M., Richardson, T. J. & Hwang, K.-T. (2004). *J. Power Sources*, **135**, 240–248.

[bb78] Drits, V. A., Lanson, B., Gaillot, A.-C. (2007). *Am. Mineral.* **92**, 771–788.

[bb15] Drits, V. A., Silvester, E., Gorshkov, A. I. & Manceau, A. (1997). *Am. Mineral.* **82**, 946–961.

[bb16] Fang, C., Huang, Y., Zhang, W., Han, J., Deng, Z., Cao, Y. & Yang, H. (2016). *Adv. Energ. Mater.* 10.1002/aenm.201501727.

[bb17] Gemmi, M. & Oleynikov, P. (2013). *Z. Kristallogr.* **228**, 51–58.

[bb18] Gorgojo, P., de la Iglesia, Ó. & Coronas, J. (2008). *Inorganic Membranes: Synthesis, Characterization and Applications*, edited by R. Mallada and M. Menéndez, pp. 135–175. Amsterdam: Elsevier.

[bb19] Grangeon, S., Lanson, B. & Lanson, M. (2014). *Acta Cryst.* B**70**, 828–838. 10.1107/S2052520614013687 25274516

[bb20] Gregorkiewitz, M. & Vezzalini, G. (1989). *Computer Aided Space Group Determination.* 12th European Crystallographic Meeting, Moscow, Collected Abstracts, Vol. 3, p. 149.

[bb21] Hammersley, A. P. (1997). *FIT2D: an Introduction and Overview.* ESRF Internal Report ESRF97HA02T.

[bb22] Henke, B. L., Gullikson, E. M. & Davis, J. C. (1993). *At. Data Nucl. Data Tables*, **54**, 181–342.

[bb23] Hu, F. & Doeff, M. M. (2004). *J. Power Sources*, **129**, 296–302.

[bb79] Jeong, Y. U. & Manthiram, A. (2001). *J. Solid. State Chem.* **156**, 331–338.

[bb24] Jiang, J., Jorda, J. L., Yu, J., Baumes, L. A., Mugnaioli, E., Diaz-Cabanas, M. J., Kolb, U. & Corma, A. (2011). *Science*, **333**, 1131–1134. 10.1126/science.1208652 21868673

[bb80] Kim, H., Kim, D. J., Seo, D.-H., Yeom, M. S., Kang, K., Kim, D. K. & Jung, Y. (2012). *Chem. Mater.* **24**, 1205–1211.

[bb25] Kolb, U., Gorelik, T., Kübel, C., Otten, M. T. & Hubert, D. (2007). *Ultramicroscopy*, **107**, 507–513. 10.1016/j.ultramic.2006.10.007 17234347

[bb26] Kolb, U., Mugnaioli, E. & Gorelik, T. E. (2011). *Cryst. Res. Technol.* **46**, 542–554.

[bb27] Kruk, I., Zajdel, P., van Beek, W., Bakaimi, I., Lappas, A., Stock, C. & Green, M. A. (2011). *J. Am. Chem. Soc.* **133**, 13950–13956. 10.1021/ja109707q 21800890

[bb28] La Mantia, F., Pasta, M., Deshazer, H. D., Logan, B. E. & Cui, Y. (2011). *Nano Lett.* **11**, 1810–1813. 10.1021/nl200500s 21413685

[bb29] Lan, C., Gong, J., Liu, S. & Yang, S. (2011). *Nanoscale Res. Lett.* **6**, 133. 10.1186/1556-276X-6-133 PMC3211180 21711626

[bb30] Lanson, B., Drits, V. A., Feng, Q. & Manceau, A. (2002). *Am. Mineral.* **87**, 1662–1671.

[bb31] Larson, A. C. & Von Dreele, R. B. (2004). *GSAS.* Report LAUR 86–748. Los Alamos National Laboratory, New Mexico, USA.

[bb32] Lee, J., Kim, S., Kim, C. & Yoon, J. (2014). *Energy Environ. Sci.* **7**, 3683–3689.

[bb33] Li, Y. & Wu, Y. (2009). *Nano Res.* **2**, 54–60.

[bb34] Liu, S., Fan, C.-Z., Zhang, Y., Li, C.-H. & You, X.-Z. (2011). *J. Power Sources*, **196**, 10502–10506.

[bb35] Martínez-Franco, R., Moliner, M., Yun, Y., Sun, J., Wan, W., Zou, X. & Corma, A. (2013). *Proc. Natl Acad. Sci. USA*, **110**, 3749–3754. 10.1073/pnas.1220733110 PMC3593851 23431186

[bb36] McCusker, L. B. & Baerlocher, C. (2009). *Chem. Commun.* pp. 1439–1451. 10.1039/b821716e 19277355

[bb37] Meier, W. M. & Villiger, H. (1969). *Z. Kristallogr.* **129**, 411–423.

[bb38] Merlini, M. & Hanfland, M. (2013). *High. Press. Res.* **33**, 511–522.

[bb39] Momma, K. & Izumi, F. (2011). *J. Appl. Cryst.* **44**, 1272–1276.

[bb40] Mugnaioli, E. (2015). *Fis. Acc. Lincei*, **26**, 211–223.

[bb41] Mugnaioli, E., Gorelik, T. & Kolb, U. (2009). *Ultramicroscopy*, **109**, 758–765. 10.1016/j.ultramic.2009.01.011 19269095

[bb42] Mugnaioli, E. & Kolb, U. (2013). *Microporous Mesoporous Mater.* **166**, 93–101.

[bb43] Mumme, W. G. (1968). *Acta Cryst.* B**24**, 1114–1120.

[bb44] Nam, K. W., Kim, S., Yang, E., Jung, Y., Levi, E., Aurbach, D. & Choi, J. W. (2015). *Chem. Mater.* **27**, 3721–3725.

[bb45] Palatinus, L. (2011). *PETS – Program for Analysis of Electron Diffraction Data.* Institute of Physics of the AS CR, Prague, Czech Republic.

[bb46] Palatinus, L. & Chapuis, G. (2007). *J. Appl. Cryst.* **40**, 786–790.

[bb47] Palatinus, L., Corrêa, C. A., Steciuk, G., Jacob, D., Roussel, P., Boullay, P., Klementová, M., Gemmi, M., Kopeček, J., Domeneghetti, M. C., Cámara, F. & Petříček, V. (2015). *Acta Cryst.* B**71**, 740–751. 10.1107/S2052520615017023 26634732

[bb48] Palatinus, L., Jacob, D., Cuvillier, P., Klementová, M., Sinkler, W. & Marks, L. D. (2013). *Acta Cryst.* A**69**, 171–188. 10.1107/S010876731204946X 23403968

[bb49] Palatinus, L., Petříček, V. & Corrêa, C. A. (2015). *Acta Cryst.* A**71**, 235–244. 10.1107/S2053273315001266 25727873

[bb50] Parant, J.-P., Olazcuaga, R., Devalette, M., Fouassier, C. & Hagenmuller, P. (1971). *J. Solid State Chem.* **3**, 1–11.

[bb51] Pasero, M. (2005). *Rev. Mineral. Geochem.* **57**, 291–305.

[bb52] Petříček, V., Dušek, M. & Palatinus, L. (2014). *Z. Kristallogr.* **229**, 345–352.

[bb53] Post, J. E. & Veblen, D. R. (1990). *Am. Mineral.* **75**, 477–489.

[bb54] Raekelboom, E. A., Hector, A. L., Owen, J., Vitins, G. & Weller, M. T. (2001). *Chem. Mater.* **13**, 4618–4623.

[bb55] Ramachandran, G. N. & Srinivasan, R. (1959). *Acta Cryst.* **12**, 410–411.

[bb56] Richardson, T. J., Ross, P. N. Jr & Doeff, M. M. (1998). *XRD Study of Lithium Insertion/Extraction in Cathodes Derived from Na_0.44_MnO_2_.* Proceedings of the Symposium on Lithium Batteries, Electrochemical Society **16**, 229–236.

[bb57] Rozhdestvenskaya, I., Mugnaioli, E., Czank, M., Depmeier, W., Kolb, U., Reinholdt, A. & Weirich, T. (2010). *Mineral. Mag.* **74**, 159–177.

[bb81] Sauvage, F., Laffont, L., Tarascon, J.-M. & Baudrin, E. (2007). *Inorg. Chem.* **46**, 3289–3294. 10.1021/ic0700250 17375916

[bb58] Shannon, R. D. (1976). *Acta Cryst.* A**32**, 751–767.

[bb59] Sheldrick, G. M. (2008). *Acta Cryst.* A**64**, 112–122. 10.1107/S0108767307043930 18156677

[bb60] Shen, X., Ding, Y., Liu, J., Laubernds, K., Zerger, R. P., Polverejan, M., Son, Y.-C., Aindow, M. & Suib, S. L. (2004). *Chem. Mater.* **16**, 5327–5335.

[bb61] Stephens, P. W. (1999). *J. Appl. Cryst.* **32**, 281–289.

[bb62] Suib, S. L. (2008). *Acc. Chem. Res.* **41**, 479–487. 10.1021/ar7001667 18232663

[bb63] Tasci, E. S., de la Flor, G., Orobengoa, D., Capillas, C., Perez-Mato, J. M. & Aroyo, M. I. (2012).* EPJ Web of Conferences* **22**, 00009; http//dx..org/10.1051/epjconf/20122200009.

[bb64] Thompson, P., Cox, D. E. & Hastings, J. B. (1987). *J. Appl. Cryst.* **20**, 79–83.

[bb82] Tian, P. & Billinge, S. J. L. (2011). *Z. Kristallogr.* **226**, 898–904.

[bb65] Toby, B. H. (2001). *J. Appl. Cryst.* **34**, 210–213.

[bb66] Tompsett, D. A. & Islam, M. S. (2013). *Chem. Mater.* **25**, 2515–2526.

[bb67] Tsuda, M., Arai, H., Nemoto, Y. & Sakurai, Y. (2001). *J. Power Sources*, **102**, 135–138.

[bb68] Tsuda, M., Arai, H., Nemoto, Y. & Sakurai, Y. (2003). *J. Electrochem. Soc.* **150**, A659–A664.

[bb69] Turner, S. & Post, J. E. (1988). *Am. Mineral.* **73**, 1155–1161.

[bb70] Vincent, R. & Midgley, P. A. (1994). *Ultramicroscopy*, **53**, 271–282.

[bb71] Wadsley, A. D. (1953). *Acta Cryst.* **6**, 433–438.

[bb72] Wang, Y., Mu, L., Liu, J., Yang, Z., Yu, X., Gu, L., Hu, Y.-S., Li, H., Yang, X.-Q., Chen, L. & Huang, X. (2015). *Adv. Energ. Mater.* **5**, 1501005.

[bb73] Wei, W., Cui, X., Chen, W. & Ivey, D. G. (2011). *Chem. Soc. Rev.* **40**, 1697–1721. 10.1039/c0cs00127a 21173973

[bb74] Yabuuchi, N. & Komaba, S. (2014). *Sci. Technol. Adv. Mater.* **15**, 043501. 10.1088/1468-6996/15/4/043501 PMC5090682 27877694

[bb75] Zhang, D., Oleynikov, P., Hovmöller, S. & Zou, X. (2010). *Z. Kristallogr.* **225**, 94–102.

