# Supplementary material for: (Na,□)5[MnO2]13 nanorods: a new tunnel structure for electrode materials determined ab initio and refined through a combination of electron and synchrotron diffraction data
Source: Acta Crystallogr B Struct Sci Cryst Eng Mater. 2016 Dec 1;72(Pt 6):893–903. doi: 10.1107/S2052520616015651 (PMC5134763; doi:10.1107/S2052520616015651)
Supplement: Supplementary file 5 [file b-72-00893-sup5.pdf]

STRUCTURAL SCIENCE
CRYSTAL ENGINEERING
MATERIALS

Volume 72 (2016)

Supporting information for article:

(Na,□)5[MnO2]13 nanorods: a new tunnel structure for electrode materials determined *ab initio* and refined through a combination of electron and synchrotron diffraction data

Enrico Mugnaioli, Mauro Gemmi, Marco Merlini and Michele Gregorkiewitz

S1. Experimental methods

S1.1. Details for laboratory X-ray powder diffraction data

Laboratory X-ray diffraction patterns were obtained using a Panalytical X'pert powder diffractometer with Bragg-Brentano geometry, Ni-filtered CuK$_{\alpha}$ radiation (λ= 1.5405981 and 1.5444183 Å) and an X'Celerator linear position sensitive detector. Axial divergence was defined by 2*0.04 rad Soller slits in the incident and the diffracted beam, divergence in the diffraction plane was 0.5º, and resolution on the Seemann-Bohlin parafocusing circle was ~0.1 mm. The powder was placed in a Si(100) single crystalline sample holder in order to minimize background, and data were collected in θ-2θ scans from 2θ = 3º to 120º (d= 29 to 0.89 Å) at 0.017º (2θ) step intervals.

S2. Results, Synthesis and composition

S2.1. Powder diffraction patterns

Figure S1 shows the powder X-ray diffraction patterns for $(Na_x\square_{1-x})_5[MnO_2]_{13}$, x=0.80, as obtained on a laboratory diffractometer and, 6 months later, with synchrotron radiation. In the meantime, hydration of the impurity phase $Na_2Mn_3O_7$ toward $(Na,H_2O)(Mn,\square)O_2$, birnessite, had proceeded and the crystallinity of birnessite increased as discussed in Section S3.3.2. At higher angles, the signal to noise ratio of the laboratory pattern is low (not shown here) whereas the synchrotron pattern contains very well defined peaks (Figure 4).

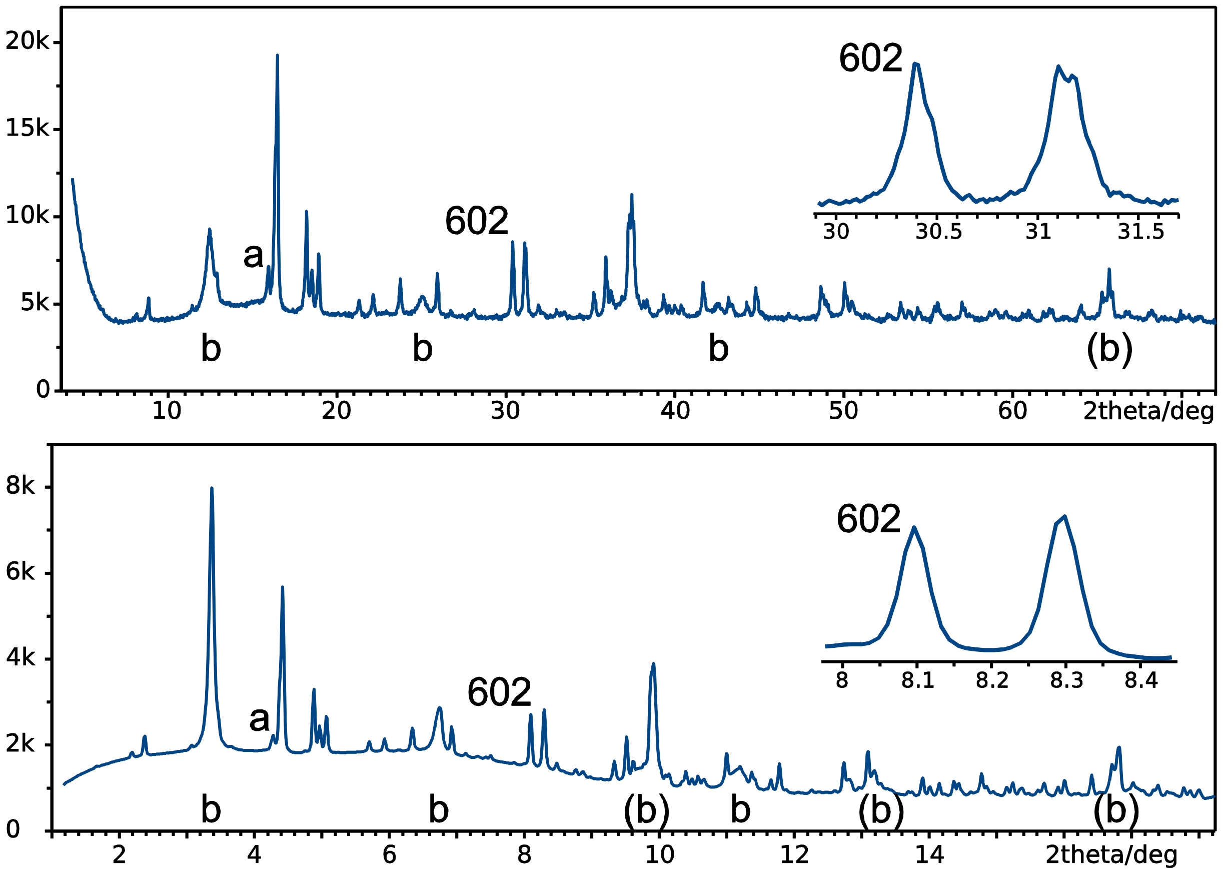

Figure S1 Powder X-ray diffraction patterns for $(Na_x\square_{1-x})_5[MnO_2]_{13}$, x=0.80. (top) Laboratory diffractometer with Bragg-Brentano geometry and CuK_α radiation, (bottom) synchrotron setup with 0.2 mm glass capillary, flat 2D detector and λ= 0.415352 Å. Labelled peaks are mainly (partly) due to the impurity phases birnessite (**b**, ~40%) and $Na_2Mn_3O_7$ (**a**, ~2%). Reflection 602 (title compound) has no overlap and can be used to assess *FWHM*. Note that the SR pattern has been obtained six months later, for the same sample.

S3. Results, Crystal structure model from single crystal electron diffraction intensities

Table S1 Summary of the parameters for EDT intensity data collection and agreement factors for the structure solutions obtained by SIR2011 (Burla *et al.*, 2012) and Jana2006 (Petříček *et al.*, 2014). U_{iso} is the average atomic displacement parameter obtained through a Wilson plot by SIR2011. The R_{val} given by Jana2006 is obtained taking the structure model derived by Superflip and refining, in kinematical approximation, only the scale factor. The last four lines give the conventional residuals and goodness of fit for least squares refinements in kinematical (framework + two Na sites; SHELX97) (Sheldrick, 2008) and dynamical (full model, JANA2006) (Petříček *et al.*, 2014; Palatinus *et al.*, 2013;Palatinus *et al.*, 2015a; Palatinus *et al.*, 2015b) approach.

Tilt range (°)	110
Number of patterns	111
Precession angle (°)	1
Reflections unique/total	843/2237
Completeness to d=0.8 Å	0.74
$R_{sym}(I)$ for $C1 \rightarrow C2/m$	0.135
$R_{\sigma}(I)=\sum\sigma I/\sum I$ (NR=843)	0.157
U_{iso} (pm^2)	118
$R_{val}(I)$ from SIR	0.226
$R_{val}(I)$ obs/all from JANA	0.27/0.32
$R1(F)$ kinematical (NP=55, NR=360/843)	0.171/0.263
$GooF$ kinematical (NP=55, NR=843)	1.89
$R(F)$ dynamical (NP=206, NR=2454/6797)	0.067/0.239
$GooF$ dynamical (NP=206, NR=2454/6797)	2.10/1.49

S4. Results, Structure refinement

S4.1. Limits of resolution of laboratory X-ray diffraction data

Rietveld refinement against laboratory X-ray powder diffraction data confirmed and improved the EDT unit cell. Nonetheless, the refinement of atom positions for the lighter elements (O, Na) was unstable and obliged to introduce "soft constraints" for the Mn-O distance using the Distance Least Squares (DLS) method (Meier &Villiger, 1969) with *d*(Mn-O)=1.9(2) Å as reference value. The restrained model converged to χ^2=8.47, R_{wp}=0.023, R_p=0.016 and R_{F^2}=0.10 for *NY*=3599 observations, *NR*=1040 reflections and *NP*=65 refined parameters, showing that X-ray intensities support the model derived from EDT data, but except for the unit cell, no improved parameters could be obtained, in particular regarding the positions and site occupation factors of sodium in the different channels.

The problem was probably due to poor peak resolution and can be rationalized as follows. From the reciprocal unit cell volume alone one would expect that the density of peaks $\partial NR/\partial(2\theta)$ exceeds $1/FWHM$ at about 70º(2θ). Calculations had therefore been limited to $2\theta \leq 70$º, but in our case, $\partial NR/\partial(2\theta)$ is irregularly distributed in the reciprocal space as a consequence of the highly anisometric unit cell dimensions. Reflections appear therefore in tight groups, separated by free spaces, even at low angles where the most prominent peaks are located (Figures S1 and 4).

S4.2. Details of refinement using synchrotron radiation diffraction data

Efforts have been made to find the most adequate model for the birnessite impurity checking the various stacking polytypes described in Drits *et al.* (2007). In our sample, birnessite is not a residue of the starting material, which is hausmannite, but it must form by decomposition of $Na_2Mn_3O_7$, which forms at high temperatures (Chang & Jansen, 1985; Raekelboom *et al.*, 2001) and may then hydrate (Parant *et al.*, 1971; Chen *et al.*, 1996; Caballero *et al.*, 2002; Nam *et al.*, 2015), during washing after the second step in the preparation of $(Na,\square)_5[MnO_2]_{13}$ (see Experimental Methods). In fact, comparison of the two patterns in Figure S1 shows that, six months after the synthesis, the birnessite peaks at 12.5º and 25.2º (2θ, CuK_α) had become much narrower indicating progressive increase in crystallinity while some of the parent $Na_2Mn_3O_7$ (peak **a** in Figure S1, actually very few) might still have transformed to birnessite. In this situation, we expect a disordered structure which might differ from that of the classical birnessite obtained in aqueous chemistry. In the final stage of refinement, eight structural parameters of birnessite had therefore been allowed to refine. The resulting model (R_{F^2}= 0.018, Table 1) shows only small differences with respect to Post & Veblen (1990), except for the *a* cell parameter which is clearly shorter (4.951 Å, Table 1) than the reference value (5.174 Å) (Post & Veblen, 1990). This is compatible with the derivation of our birnessite from $Na_2Mn_3O_7$, as the

in-plane dimensions of their octahedral sheet (7.07 and 7.05 Å^2/Mn, respectively) resemble each other and are significantly smaller than for ordinary birnessite (7.37 Å^2/Mn) (Post & Veblen, 1990). A more detailed study of this aspect would be interesting but has to wait for better defined and phase pure birnessite samples.
SR data allowed to refine more parameters (123 instead of 65) and the improvement owes certainly much to the increased number of background and profile parameters (+31). Several tests have therefore been conducted to corroborate the soundness of the final model, in particular with regard to the Mn^{3+}-Mn^{4+} distribution and the Na3 position.

The relevance of the Mn^{3+}-Mn^{4+} distribution was checked comparing models with free vs restraint Mn-O distances. Keeping constant the number of refinable parameters (*NP*=123) and starting from the final model (Tables 1 and S2), we successively increased the relative weight of the Mn-O distance restraints, *DLSF*, from 0 to 10k and allowed the model to adapt until convergence. The corresponding residuals, calculated for the series *DLSF*= 0-1-10-100-1k-10k, were R_{F2}= 0.0350-0.0350-0.0355-0.0388-0.0486-0.0588. The model with *DLSF*=1k, the factor necessary for a stable refinement with laboratory data, is considerably worse (0.0486 *vs.* 0.0350), *i.e.* the release from a model with all octahedra the same size (1.895(38) Å) to a more differentiated model (1.930(77) Å, see also Table S4) with an ordered Mn^{3+}-Mn^{4+} distribution is highly significant.

Another critical parameter is the Na3 position which, during model solution, appeared as a small peak in the six-ring channel (Figure 3) visible in difference Fourier syntheses. There are two six-ring channels per unit cell but the possible Wyckoff site here has multiplicity 4 (special coordinates and site symmetry $\bar{1}$, Table S2), *i.e.* each channel has two positions per unit cell, at a distance *d*(Na3-Na3)=*b*/2=1.42 Å. This is shorter than twice the ionic radius of Na^+ (1.02 Å) (Shannon, 1976) and the two positions can only be partially occupied (site occupation factor *SOF*≤0.5) forcing a statistical distribution which comes near to an undisrupted chain along **b** (Figure 5). An alternative site, again with multiplicity 4 and displaced along the tunnel by *b*/4 (*i.e.* *y*=0), was tested and gave almost indistinguishable results (Table S4), with slightly better reliability indices (χ^2=0.680, R_{F2}=0.0347 instead of χ^2=0.690, R_{F2}=0.0350) but one unusually short Na-O distance (2.07 Å). We therefore preferred the former model, keeping in mind that it describes a strongly displaced atom.

This also poses the question of a possible symmetry descent to one of the subgroups of *C2/m*. Lowering the symmetry, both $C\bar{1}$ and *Cm* offer a Na position with multiplicity 2 in the six-ring channel. Several trials for symmetry release were undertaken but they all run unavoidably into problems with parameter correlations and indicated no way to improve the model. Considering the low diffraction power of Na and the fact that most of the discrepancies are due to the presence of

birnessite, a descent to subgroups can therefore be ruled out at the present (actually rather advanced) stage of refinement.

Note also that the powder residuals (R_{wp}=0.051, R_p=0.037) are higher than with laboratory data (R_{wp}=0.023, R_p=0.016), a usual observation when passing from low resolution to crisper diffraction patterns.

Table S2 Atom parameters obtained for $(Na_x□_{1-x})_5[MnO_2]_{13}$, *x*=0.80(4), space group *C12/m1*, from Rietveld refinement (22.5199(6) 2.83987(6) 14.8815(4) Å, β=105.0925(16)º, *Z*=2) and EDT dynamical refinement (22.6338 2.8255 14.9075 Å, β= 104.5992º, *Z*=2). *Mu-Wy-Sy* give the multiplicity, Wyckoff notation and point symmetry. Standard deviations (in parentheses) refer to last digits.

Rietveld refinement						
atom	*Mu Wy Sy*	*x*	*y*	*z*	*SOF*	U_{iso}/pm^2
Mn1	2 *b 2/m*	0.5	0	0	1	93(19)
Mn2	4 *i m*	0.3931(2)	0.5	0.0258(3)	1	59(14)
Mn3	4 *i m*	0.3439(2)	0	0.2042(3)	1	30(13)
Mn4	4 *i m*	0.4311(2)	0.5	0.3632(3)	1	57(12)
Mn5	4 *i m*	0.5795(2)	0	0.4308(3)	1	80(13)
Mn6	4 *i m*	0.6596(2)	0.5	0.3380(3)	1	101(14)
Mn7	4 *i m*	0.7078(2)	0	0.1773(3)	1	63(12)
O1	4 *i m*	0.4544(7)	0	0.0910(12)	1	82(13)
O2	4 *i m*	0.5533(7)	0.5	0.0573(11)	1	= *U*(O1)
O3	4 *i m*	0.6565(7)	0	0.0319(11)	1	= *U*(O1)
O4	4 *i m*	0.3536(8)	0.5	0.1220(13)	1	= *U*(O1)
O5	4 *i m*	0.2557(7)	0	0.1473(11)	1	= *U*(O1)
O6	4 *i m*	0.3355(7)	0.5	0.2885(11)	1	= *U*(O1)
O7	4 *i m*	0.4239(7)	0	0.2624(12)	1	= *U*(O1)
O8	4 *i m*	0.5247(7)	0.5	0.3932(11)	1	= *U*(O1)
O9	4 *i m*	0.4233(7)	0	0.4429(11)	1	= *U*(O1)
O10	4 *i m*	0.6418(7)	0.5	0.4539(11)	1	= *U*(O1)
O11	4 *i m*	0.5999(7)	0	0.3117(12)	1	= *U*(O1)
O12	4 *i m*	0.6647(7)	0.5	0.2160(11)	1	= *U*(O1)
O13	4 *i m*	0.7146(8)	0	0.3735(11)	1	= *U*(O1)
Na1	4 *i m*	0.3017(8)	0	0.4119(13)	0.808(19)	660(70)
Na2	4 *i m*	0.5095(8)	0.5	0.2226(12)	0.816(20)	= *U*(Na1)
Na3	4 *e -1*	0.25	0.25	0	0.368(12)	= *U*(Na1)

Table S2. (*cont.*)

EDT dynamical refinement						
atom	*Mu Wy Sy*	*x*	*y*	*z*	*SOF*	U_{iso} or U_{ani}/pm^2
Mn1	2 *b 2/m*	0.5	0	0	1	173(14)
Mn2	4 *i m*	0.39251(12)	0.5	0.02526(20)	1	164(10)
Mn3	4 *i m*	0.34546(13)	0	0.2073(2)	1	225(10)
Mn4	4 *i m*	0.43168(13)	0.5	0.36179(19)	1	171(10)
Mn5	4 *i m*	0.57832(13)	0	0.43259(19)	1	167(10)
Mn6	4 *i m*	0.65796(13)	0.5	0.33754(20)	1	221(10)
Mn7	4 *i m*	0.70666(13)	0	0.1789(2)	1	250(11)
O1	4 *i m*	0.4482(3)	0	0.0829(5)	1	144(13)
O2	4 *i m*	0.5517(3)	0.5	0.0599(5)	1	238(14)
O3	4 *i m*	0.6575(3)	0	0.0330(5)	1	276(15)
O4	4 *i m*	0.3536(3)	0.5	0.1294(5)	1	209(13)
O5	4 *i m*	0.2595(4)	0	0.1581(6)	1	349(16)
O6	4 *i m*	0.3399(3)	0.5	0.2904(5)	1	263(14)
O7	4 *i m*	0.4316(3)	0	0.2736(5)	1	248(14)
O8	4 *i m*	0.5216(3)	0.5	0.4004(5)	1	232(14)
O9	4 *i m*	0.4225(3)	0	0.4393(5)	1	242(14)
O10	4 *i m*	0.6398(3)	0.5	0.4601(5)	1	185(13)
O11	4 *i m*	0.5960(3)	0	0.3138(5)	1	156(12)
O12	4 *i m*	0.6591(3)	0.5	0.2143(5)	1	344(16)
O13	4 *i m*	0.7138(3)	0	0.3620(5)	1	338(16)
Na1	4 *i m*	0.3011(6)	0	0.4110(8)	0.749(18)	890(60)
Na2	4 *i m*	0.5130(6)	0.5	0.2166(9)	0.83(2)	920(60)
Na3	4 *e -1*	0.25	0.25	0	0.389(17)	1440(160)
atom	U_{11}/pm^2	U_{22}/pm^2	U_{33}/pm^2	U_{12}/pm^2	U_{13}/pm^2	U_{23}/pm^2
Mn1	260(30)	100(13)	140(20)	0	10(20)	0
Mn2	172(17)	141(10)	210(17)	0	106(17)	0
Mn3	320(2)	59(8)	239(18)	0	-28(17)	0
Mn4	246(19)	89(9)	183(16)	0	63(16)	0
Mn5	265(19)	99(9)	116(15)	0	8(16)	0
Mn6	262(19)	98(10)	282(18)	0	30(18)	0
Mn7	330(20)	104(9)	293(18)	0	34(18)	0
Na1	990(120)	870(80)	570(100)	0	-230(100)	0
Na2	740(100)	1550(110)	590(90)	0	400(100)	0

S5. Discussion, Charge ordering and Na coordination

Table S3 Interatomic distances for $(Na_x\square_{1-x})_5[MnO_2]_{13}$, x=0.79(2) as obtained from dynamical refinement using EDT intensity data. Figures in parentheses refer to last digits and have the meaning of standard deviations obtained from least squares refinement for individual, and dispersions obtained from averaging over one or more polyhedra for mean distances. For global means, Mn2, Mn4 and Mn7 are considered as Mn^{3+}.

		d/Å			*d*/Å			*d*/Å
Mn1	O1 x2	1.906(8)	Mn2	O1 x2	1.944(5)	Mn3	O4 x2	1.868(5)
	O2 x4	1.907(4)		O2	2.003(9)		O5	1.899(8)
				O3 x2	1.880(5)		O6 x2	1.904(6)
				O4	1.968(9)		O7	1.951(7)
	mean	1.907(1)		mean	1.937(49)		mean	1.899(31)
Mn4	O6	2.082(7)	Mn5	O8 x2	1.888(5)	Mn6	O10	1.972(8)
	O7 x2	1.929(5)		O9	1.915(8)		O11 x2	1.960(5)
	O8	1.970(7)		O10 x2	1.952(5)		O12	1.843(9)
	O9 x2	1.869(6)		O11	1.912(8)		O13 x2	1.869(5)
	mean	1.941(79)		mean	1.918(29)		mean	1.912(58)
Mn7	O3	2.179(8)				24x	<Mn^{4+}-O>	1.909(34)
	O5 x2	1.925(6)				17x	<Mn^{3+}-O>	1.950(79)
	O12 x2	1.929(6)				41x	<<Mn-O>>	1.926(60)
	mean	1.977(113)						
Na1	O6 x2	2.607(14)	Na2	O1 x2	2.577(11)	Na3	O3 x2	2.377(8)
	O9	2.674(15)		O2	2.694(17)		O4 x2	2.727(6)
	O10 x2	2.487(11)		O7 x2	2.627(15)		O5 x2	2.418(8)
	O13 x2	2.389(12)		O8	2.699(15)			
				O11 x2	2.501(11)			
	mean	2.52(11)		mean	2.60(8)		mean	2.51(17)

All means differ by < 1 esd from the corresponding values obtained from Rietveld refinement (Table 2). A possibly significant difference regards Mn2 where the individual Mn-O distances along O2-Mn2-O4 comply with a Jahn-Teller distortion which is not visible in Table 2. From dynamical refinement, the Mn2 site appears therefore as a third candidate to host Mn^{3+}.

S6. Discussion, Chemical formula and preferred compositions

S6.1. The Mumme framework

For the Mumme (1968) structure, the chemical formula can be given as $(Na_x\square_{1-x})_6[MnO_2]_9$ = (Na, $\square)_{0.67}[MnO_2]$. From synthesis, the material is usually obtained with a ratio *Na/Mn* = 0.4-0.5 (Doeff *et al.*, 1996; Jeong & Manthiram, 2001; Sauvage *et al.*, 2007; Akimoto *et al.*, 2011; Chu *et al.*, 2011; Kruk *et al.*, 2011), *i.e.* the average occupation of tunnel sites is *x*~ 0.70, or 8 Na per unit cell (4 Na per formula) which, in striking analogy with our case, exactly matches the number of Mn^{3+} resulting from an ordered occupation of two 4-fold Mn sites while the remainder (two 4-fold plus one 2-fold Mn site) is Mn^{4+}.

If ordered Mn^{3+}-Mn^{4+} distribution was the case throughout, we would expect a preference for degrees of filling at *Na/Mn*= 0, 0.22, 0.44, 0.67 (0, 4, 8, 12 Na per unit cell). Experiment (electrochemical behaviour and chemical oxidation) tells us that the actually accessible range of compositions, in the Mumme (1968) framework, is limited to 0.2<*Na/Mn*<0.7 (Doeff *et al.*, 1996; Armstrong *et al.*, 1998; Doeff *et al.*, 2004; Sauvage *et al.*, 2007; Kim *et al.*, 2012), and the corresponding crystal structures (Mn-O distances) show that Mn^{3+} is found in the square pyramid (site Mn4) for all compositions with *Na/Mn* ≥ 0.2, and on site Mn5 for *Na/Mn* ≥ 0.4 (Doeff *et al.*, 1996; Armstrong *et al.*, 1998; Richardson *et al.*, 1998; Doeff *et al.*, 2004). Density functional theory simulations (Kim *et al.*, 2012) confirm this scheme and suggest that, in the fully reduced framework (*Na/Mn*=0.67), the third Mn^{3+} is located at site Mn3 (Doeff-Armstrong-Kruk notation for site numbering). A modulated Mn^{3+}-Mn^{4+} distribution, *e.g.* along the tunnel axis and correlated with Na^+- distribution, can be expected for intermediate compositions, but has also been discussed for $Na_{0.40}[MnO_2]$ (Kruk *et al.*, 2011).

S7. Discussion, Reliability of results from EDT single crystal and X-ray powder diffraction

S7.1. Details and Table S4

When comparing structural results from different sources, the first control which comes to mind are of course the Bragg residuals (R_F and R_{F^2}) and the parameter uncertainties estimated from least squares refinement. Unfortunately, when dealing – as in the present case – with results obtained from different types of observed data, the comparison of Bragg residuals has only qualitative value.

More significant are positional uncertainties which were found to decrease systematically in the order EDT kinematical – SR Rietveld – EDT dynamical refinement, with values of 1.7-0.5-0.3 pm for manganese (3.3-1.7-0.9 for oxygen and 7.1-1.8-1.4 for sodium). Clearly, dynamical refinement gives better results than the kinematical approximation, but comparison with the results from Rietveld refinement is difficult (the theory biased laboratory Rietveld results were not considered in this context).

We therefore tried to get independent information about the reliability using the discrepancies between different models. Discrepancies for atom positions are already described in the main text, here we present the discrepancies of the framework geometry (Table S4), a derived parameter which can easily be checked from crystallochemical knowledge.

The first block in Table S4 shows the raw model obtained from EDT data using the kinematical approximation. The framework is given to surprising detail: Mn-O distances are only slightly smaller than in the final model (191 pm instead of 193 pm) and even some Mn^{3+}-Mn^{4+} order with Mn^{3+} on (Mn2-)Mn4-Mn7 can be recognized. However, random dispersion of Mn-O distances is high and the minimum values of <185 pm are unreasonable.

The results for laboratory X-ray powder data (third block) clearly reflect the application of DLS restraints (factor *DLSF*=1000) which obliged the Mn-O distances to cluster around a mean value (189 pm) with very little dispersion (2 pm), about half of the dispersion found in the other cases. Nevertheless, there are still some quite small minimum distances (Mn5, Mn6, Mn7), and the octahedral angles, not restrained by DLS, show the highest variance among all models in Table S4 (117 vs ~36 deg^2).

Table S4 Synoptic presentation of some structural results obtained for refinements based on different observed data, to evidence the corresponding reliability. Mean for *qelong*=quadratic elongation is given for Mn1-Mn3-Mn5-Mn6, *i.e.* the octahedra containing only Mn^{4+} (no Jahn-Teller effect). *lin dis* and *ang dis* are the (linear) distance distortion index and the angle variance (deg^2), respectively. Values calculated with the aid of VESTA (Momma & Izumi, 2011).

electron single crystal, kinematical, χ^2_{all}*= 3.57,* $R1(F)_{all}$*= 0.263,* $R1(F)_{obs}$ *= 0.171*							
Mn#	1	2	3	4	5	6	7	mean
<Mn-O>	185	196	190	193	189	191	195	191(4)
Mn-Omin	182	191	184	184	188	181	187	
Mn-Omax	187	202	199	213	189	201	212	
lin dis	.013	.019	.022	.040	.003	.034	.036	.024(13)
qelong	1.014	1.015	1.013	1.019	1.008	1.013	-	1.012(3)
ang dis	43	48	40	48	27	39	-	41(8)
CNe/CN	6.0/6	5.9/6	5.8/6	5.5/6	6.0/6	5.7/6	4.6/5	5.8(2)/6
electron single crystal, dynamical, χ^2_{all}*= 2.22,* R_{Fall}*= 0.239,* R_{Fobs} *= 0.067*								
Mn#	1	2	3	4	5	6	7	mean
<Mn-O>	190.7	193.7	189.9	194.1	191.8	191.2	197.7	192.7(2.7)
Mn-Omin	190.6	188.0	186.8	186.9	188.8	184.3	192.5	
Mn-Omax	190.7	200.3	195.1	208.2	195.2	197.2	217.9	
lin dis	.0002	.019	.011	.029	.012	.027	.041	.020(14)/6
qelong	1.011	1.011	1.009	1.015	1.007	1.010	-	1.009(2)
ang dis	36	35	29	42	22	31	-	33(7)
CNe/CN	6.0/6	5.9/6	6.0/6	5.7/6	6.0/6	5.8/6	4.6/5	5.9(1)/6
X'pert Rietveld, DLSF=1000, χ^2*= 8.47,* R_{F^2}*= 0.10*								
Mn#	1	2	3	4	5	6	7	mean
<Mn-O>	188	193	192	189	189	186	189	189(2)
Mn-Omin	187	188	189	186	183	185	184	
Mn-Omax	189	198	197	192	193	187	199	
lin dis	.006	.020	.013	.011	.014	.005	.022	.013(6)
qelong	1.062	1.039	1.042	1.031	1.020	1.033	-	1.039(18)
ang dis	178	124	142	93	64	99	-	117(40)
CNe/CN	6.0/6	5.9/6	6.0/6	6.0/6	5.9/6	6.0/6	4.8/5	5.97(5)/6
SR Rietveld, y(Na3)=¼, χ^2*= 0.690,* R_{F^2}*=0.0350*								
Mn#	1	2	3	4	5	6	7	mean
<Mn-O>	190.8	193.9	190.8	200.7	191.7	188.4	195.3	193.1(4.0)
Mn-Omin	190.1	187.1	178.3	188.7	186.9	184.9	189.3	
Mn-Omax	191.1	204.1	194.7	215.0	196.2	192.4	217.2	
lin dis	.002	.035	.022	.040	.020	.014	.045	.025(15)
qelong	1.008	1.011	1.004	1.022	1.012	1.009	-	1.008(3)
ang dis	27	31	12	62	40	31	-	34(17)
CNe/CN	6.0/6	5.7/6	5.7/6	5.4/6	5.9/6	5.9/6	4.6/5	5.8(2)/6
SR Rietveld, y(Na3)=0, χ^2*= 0.680,* R_{F^2}*=0.0347*								
Mn#	1	2	3	4	5	6	7	mean
<Mn-O>	190.9	193.8	190.9	200.8	191.6	188.3	195.4	193.1(4.1)
Mn-Omin	190.2	186.9	178.0	189.6	186.7	184.3	189.4	
Mn-Omax	191.2	203.8	194.7	215.3	196.5	192.3	217.6	
lin dis	.002	.035	.022	.037	.022	.014	.046	.025(15)
qelong	1.008	1.011	1.005	1.021	1.012	1.009	-	1.009(3)
ang dis	26	31	11	62	39	31	-	33(17)
CNe/CN	6.0/6	5.7/6	5.7/6	5.5/6	5.9/6	5.9/6	4.5/5	5.8(2)/6

Note: for Rietveld refinements, χ^2=*SQER*/(*NY*-*NP*) is the reduced chi square where the sum in *SQER* = S$w_i(Y_i^{obs}-Y_i^{cal})^2$ goes over the *NY* points in the intensity profile, w_i is the weight of Y_i^{obs} and *NP* the number of refined parameters. For kinematical (SHELX97) and dynamical (JANA2006) refinements, $\chi^2 = GooF^2 = \sum w_i(I_i^{obs}-I_i^{cal})^2/(NI-NP)$ with the sum going over the *NI* reflections.

SR data (fourth block) neatly differentiate between two subsets of Mn polyhedra, the first (Mn1-Mn3-Mn5-Mn6) with a mean Mn-O distance of 190.4(1.4) pm corresponding to Mn^{4+}, and the second (Mn2-Mn4-Mn7) which contains Mn^{3+} to varying degrees and has Mn-O distances ranging from 193.9 to 200.7 pm. The Mn4 octahedron has its long axis (O6-Mn4-O8) lying in the **ac** plane as expected for Jahn-Teller distortion but for Mn2, no long axis can be recognized. From SR data, Mn^{3+} is therefore expected to occupy mainly the Mn4 and Mn7 sites. The choice of the alternative *y* coordinates for Na3 has, as discussed above, no effect on the framework (see fifth block).

The results from EDT dynamical refinement (second block) are similar to those obtained from SR data, with Mn^{4+} on sites Mn1-Mn3-Mn5-Mn6 and a mean distance of 190.8(0.9) pm. However, Mn2 shows now clearly a long axis (O2-Mn2-O4) in the **ac** plane, suggesting that, in addition to Mn7 and Mn4, also Mn2 may contain some Mn^{3+}. From standard deviations, the difference between the two hypotheses seems significant but errors might actually be higher considering that the weights of powder intensities from area detectors and samples containing impurities are difficult to handle (Tian & Billinge, 2011; David, 2001). One experimental datum in support of error underestimation is the short Mn3-O distance of 178.3(1.5) pm obtained from SR data, about 7 esd away from the Shannon (1976) reference value of 189 pm.

References

Akimoto, J., Hayakawa, H., Kijima, N., Awaka, J. & Funabiki, F. (2011). *Sol. St. Phen.* **170**, 198–202.

Armstrong, A. R., Huang, H., Jennings, R. A. & Bruce, P G. (1998). *J. Mater. Chem.* **8**, 255–259.

Burla, M. C., Caliandro, R., Camalli, M., Carrozzini, B., Cascarano, G. L., Giacovazzo, C., Mallamo, M., Mazzone, A., Polidori, G. & Spagna, R. (2012). *J. Appl. Cryst.* **45**, 357–361.

Caballero, A., Hernán, L., Morales, J., Sánchez, L., Santos Peña, J. & Aranda, M. A. G. (2002). *J. Mater. Chem.* **12**, 1142–1147.

Chang, F. M. & Jansen, M. (1985). *Z. Anorg. Allg. Chem.* **531**, 177–182.

Chen, R., Chirayil, T., Zavalij, P. & Whittingham, M. S. (1996). *Solid State Ionics* **86-88**, 1–7.

Chu, Q., Wang, X., Li, Q. & Liu, X. (2011). *Acta Cryst.* C**67**, i10–i12.

David, W. I. F. (2001). *J. Appl. Cryst.* **34**, 691–698.

Doeff, M. M., Richardson, T. J. & Kepley, L. (1996). *J. Electrochem. Soc.* **143**, 2507–2516.

Doeff, M. M., Richardson, T. J. & Hwang, K.-T. (2004). *J. Power Sources* **135**, 240–248.

Drits, V. A., Lanson, B., Gaillot, A.-C. (2007). *Am. Mineral.* **92**, 771–788.

Jeong, Y. U. & Manthiram, A. (2001). *J. Solid. State Chem.* **156**, 331–338.

Kim, H., Kim, D. J., Seo, D.-H., Yeom, M. S., Kang, K., Kim, D. K. & Jung, Y. (2012). *Chem.Mater.* **24**, 1205–1211.

Kruk, I., Zajdel, P., van Beek, W., Bakaimi, I., Lappas, A., Stock, C. & Green, M. A. (2011). *J. Am. Chem. Soc.* **133**, 13950–13956.

Meier, W. M. & Villiger, H. (1969). *Z. Kristallogr.* **129**, 411–423.

Momma, K. & Izumi, F. (2011). *J. Appl. Cryst.* **44**, 1272–1276.

Mumme, W G. (1968). *Acta Cryst.* B**24**, 1114–1120.

Nam, K. W., Kim, S., Yang, E., Jung, Y., Levi, E., Aurbach, D. & Choi, J. W. (2015). *Chem. Mater.* **27**, 3721–3725.

Palatinus, L., Jacob, D. Cuvillier, P., Klementová, M., Sinkler, W. & Marks, L. D. (2013). *Acta Cryst.* A**69**, 171–188.

Palatinus, L., Antunes Corrêa, C., Steciuk, G., Jacob, D., Roussel, P., Boullay, P., Klementová, M., Gemmi, M., Kopeček, J., Domeneghetti, M. C., Cámara, F. & Petříček, V. (2015a). *Acta Cryst.* B**71**, 740–751.

Palatinus, L., Petříček, V. & Antunes Corrêa, C. (2015b). *Acta Cryst.* A**71**, 235–244.

Parant, J.-P., Olazcuaga, R., Devalette, M., Fouassier, C. & Hagenmuller, P. (1971). *J. Solid State Chem.* **3**, 1–11.

Petříček, V., Dušek, M. & Palatinus, L. (2014). *Z. Kristallogr.* **229**, 345–352.

Post, J. E. & Veblen, D. R. (1990). *Am. Mineral.* **75**, 477–489.

Raekelboom, E. A., Hector, A. L., Owen, J., Vitins, G. & Weller, M. T. (2001). *Chem. Mater.* **13**, 4618–4623.

Richardson, T. J., Ross, P. N. Jr. & Doeff, M. M. (1998). *XRD Study of Lithium Insertion/Extraction in Cathodes Derived from* $Na_{0.44}MnO_2$*.* Proceedings of the Symposium on Lithium Batteries, Electrochemical Society 16:229-236. ISBN 9781566772105 [1566772109].

Sauvage, F., Laffont, L., Tarascon, J.-M. & Baudrin, E. (2007). *Inorg. Chem.* **46**, 3289–3294.

Shannon, R. D. (1976). *Acta Cryst.* A**32**, 751–767.

Sheldrick, G. M. (2008). *Acta Cryst.* A**64**, 112–122.

Tian, P. & Billinge, S. J. L. (2011). *Z. Kristallogr.* **226**, 898–904.
